# Mitochondrial Stress in *Helicobacter pylori* Infection and Associated Malignancies: A Review

**DOI:** 10.3390/antiox15030285

**Published:** 2026-02-25

**Authors:** Viola Varga, András Gelley, Eva Margittai, Buket Bagci, Edina Amalia Wappler-Guzzetta, Ibolya Czegle

**Affiliations:** 1 Institute of Clinical Pathophysiology, Semmelweis University, 1085 Budapest, Hungary; varga-viola.maria@semmelweis.hu (V.V.); margittai.eva@semmelweis.hu (E.M.); 2 Center of Internal Medicine, Buda Hospital of the Hospitaller Order of Saint John of God, 1023 Budapest, Hungary; gelley.andras@irgalmas.hu; 3 Department of Pathology, University of Rochester Medical Center, Rochester, NY 14642, USA; buket_bagci@urmc.rochester.edu; 4 Department of Pathology and Laboratory Medicine, Boston Medical Center and Boston University School of Medicine, Boston, MA 02118, USA; wapplerg@bu.edu; 5 Department of Internal Medicine and Hematology, Semmelweis University, 1085 Budapest, Hungary

**Keywords:** *Helicobacter pylori*, mitochondrial stress, gastric cancer, MALT lymphoma

## Abstract

*Helicobacter pylori* (*H. pylori*) infection is one of the most common bacterial infections worldwide. Its role in infection-associated cancers, such as gastric cancer and mucosa-associated lymphoid tissue (MALT) lymphoma is well known. However, mitochondrial alterations in these malignancies are less documented. Mitochondria are key organelles, maintaining cellular homeostasis under normal and pathological conditions. They regulate complex cellular processes and play a key role in carcinogenesis and cancer progression in *H. pylori*-associated malignancies. This review summarizes the role of mitochondrial stress in *H. pylori* infection, gastric cancer, and MALT lymphoma.

## 1. General Aspects of *Helicobacter pylori*-Associated Malignancies

*Helicobacter pylori* (*H. pylori*) is a spiral shaped bacterium, first identified by Robin Warren and Barry Marshall in 1982 [1]. It is recognized as one of the most common bacterial infections worldwide, affecting over 50% of the global population [2]. This gram-negative bacterium is able to colonize the gastric mucosa, as it is highly adapted to survive in acidic environment [3]. Recent comprehensive analyses showed that the global prevalence of *H. pylori* infection slightly decreased in the adult population (from ~52.6% to ~43.9% between 2015 and 2022) due to advances in hygiene in the developing countries. In contrary, the prevalence of *H. pylori* infections remained unchanged in children and adolescents in all World Health Organizations-monitored regions [4]. Its role in gastrointestinal diseases and cancer makes it a significant public health concern; long-term infection with *H. pylori* is a well-established risk factor for gastric cancer and for mucosa-associated lymphoid tissue (MALT) lymphoma. Clinically, *H. pylori* infection can be asymptomatic, particularly in the early stages of the infection, although it can mimic peptic ulcer disease. In advanced stages, patients may present with dysphagia, weight loss, vomiting, and anemia. The gold standard for diagnosis of these diseases remained the microscopic examination of gastric biopsy specimens [3,5].

Gastric cancer remains the third most common cause of cancer-related mortality worldwide, with an estimated 1 million new cases each year [6] despite the decrease in its incidence. Importantly, about 75% of distal gastric adenocarcinomas, arising from the gastric epithelium, are attributed to *H. pylori* infection. MALT lymphoma, on the other hand, develop from gastric lymphocytes. It has a lower incidence rate than gastric cancer, and *H. pylori* eradication therapy leads to disease remission in 70% of early-stage cases [7].

*H. pylori* infection leads to chronic gastric mucosal inflammation, which has a pivotal role in the progression of these malignancies. *H. pylori* infection modifies parietal cell function, leading to decreased gastric acid production that facilitates bacterial colonization and mucosal injury [8]. The inflammatory environment subsequently promotes genetic and epigenetic changes in gastric epithelial cells, contributing to neoplastic transformation. Additionally, *H. pylori* virulence factors, such as cytotoxin-associated gene A (CagA) and vacuolating cytotoxin A (VacA) disrupt normal cellular processes, further enhancing carcinogenesis. Besides affecting epithelial cells, *H. pylori* exert various effects on immune cells, such as neutrophils, macrophages, dendritic cells, and lymphocytes, leading to the release of pro-inflammatory cytokines and chemokines that recruit and further activate immune cells, amplifying the inflammatory response in the gastric mucosa. This augmented activation and proliferation of mucosal B cells can lead to DNA mutations, eventually leading to lymphomatous transformation [9].

Recently, mitochondrial homeostasis pathway targeting emerged as a promising therapeutic strategy for both of these *H. pylori*-associated malignancies [10]. Restoration of balanced mitochondrial dynamics, mitophagy induction, or disruption of cancer-specific mitochondrial metabolism have shown potential in preclinical studies, improving treatment efficacy. This review summarizes our current understanding of mitochondrial alterations in *H. pylori*-induced gastric adenocarcinoma and MALT lymphoma.

## 2. *H. pylori* Infection and Mitochondrial Stress 

The maintenance of mitochondrial homeostasis is ensured by tightly regulated mechanisms. Their major role is ATP production via oxidative phosphorylation (OXPHOS) via the mitochondrial electron transport chain. Beyond energy production, mitochondria regulate several vital cellular processes, including ROS production, calcium homeostasis, synthesis of key molecules, and regulation of apoptosis. Mitochondria have their own circular DNA (mtDNA) and they can replicate independently within the cell. To maintain mitochondrial quality control, mtDNA repairing enzymes (mtBER, mismatch repair, POLG) and mitophagy aid repairing or getting rid of dysfunctional mitochondria. Furthermore, the balance between mitochondrial fission and fusion is crucial for mitochondrial homeostasis, cell stability and survival. Fusion is important in repairing damaged mitochondria when they fuse with a healthy mitochondrion. On the other hand, fission facilitates the damaged mitochondria to be engulfed via mitophagy. Additionally, mitochondrial fission and fusion are crucial in cellular energy production adaptation, in regulating apoptosis, and controlling oxidative stress [11].

Disruption of mitochondrial homeostasis by *H. pylori* infection plays a central role in cancer cell metabolic reprogramming, apoptosis evasion, enhanced proliferative capacity, and increased metastatic potential [12]. The infection induces shifts from OXPHOS toward glycolysis, supporting the high proliferative and metastatic potential of gastric epithelial cells. *H. pylori* is also responsible for causing mutations in mtDNA, which was shown to impair mitochondrial replication and transcription, exacerbate mitochondrial dysfunction, increase ROS production, and contribute to its tumorigenic potential [13].

**Figure 1:**
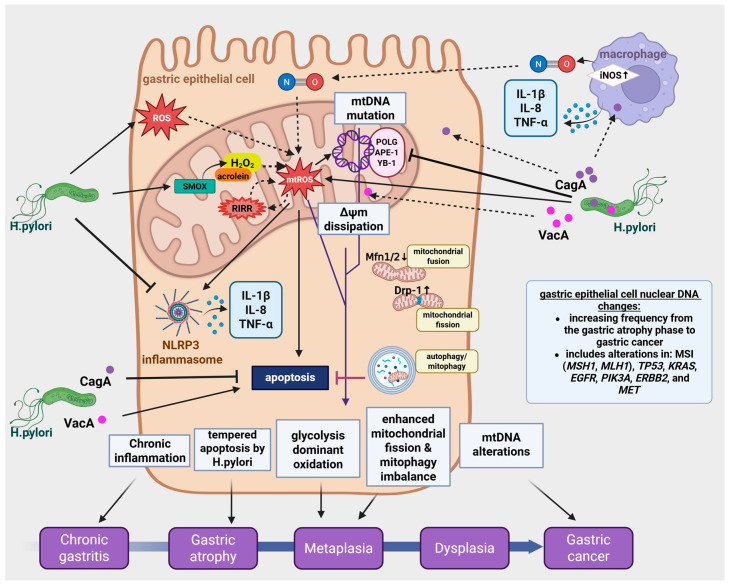
*H. pylori* infection induces multifaceted pathological changes in gastric epithelial cells, many of which are mediated via mitochondrial-related pathways. *H. pylori* mediates most of its pathogenic effects through its virulence factors, with CagA and VacA being the most crucial. VacA forms an anion-selective channel in the inner mitochondrial membrane, resulting in dissipation of the mitochondrial membrane potential (Δψm). In addition, *H. pylori* upregulates spermine oxidase (SMOX) generating H_2_O_2_ and acrolein as a byproduct. Reactive oxygen species (ROS) coming from multiple pathways from the cytoplasm together with excessive mitochondrial ROS (mtROS) production, triggering ROS-induced ROS release (RIRR), amplifying oxidative stress. Eventually, mtROS leads to mtDNA mutations, enhancing the needs for DNA repair mechanisms, such as BER or mismatch repair. However, key enzymes of DNA repair (APE-1, YB-1 and POLG) are altered upon *H. pylori* infection, which exacerbates mitochondrial DNA instability. These insults activate mitochondrial quality control pathways, including mitochondrial dynamics (fusion and fission) and mitophagy. The persistent infection and chronic inflammation will eventually favor mitochondrial fission and induce mitophagy. In later stages of the infection, these pathways will be not sufficient, and dysfunctional mitochondria will be accumulated. In addition, mitochondrial dysfunction promotes NLRP3 inflammasome activation and release of inflammatory cytokines (such as IL 1β, IL 8, and TNF α), sustaining chronic inflammation in both epithelial and immune (B-) cells. Furthermore, CagA induces iNOS expression in immune cells (such as macrophages), leading to increased production of nitric oxide (NO) that also form other reactive nitrogen species (RNS), which in turn activate downstream inflammatory signaling pathways. These mitochondrial and inflammatory perturbations converge to drive cellular transformation and metaplastic progression. In gastric epithelial cells, multiple mitochondrial quality control pathways are initially activated in response to *H. pylori* infection; however, when ROS production exceeds these compensatory defenses, mitochondria typically trigger apoptosis, often associated with excessive mitochondrial fission. At the same time, *H. pylori* can dampen NLRP3 inflammasome activation to support cell survival, while promoting a shift from OXPHOS to glycolysis, and fostering the accumulation of mtDNA mutations. Taken together, these interconnected processes steer gastric epithelial cells toward metaplasia and dysplasia, ultimately facilitating malignant transformation during persistent *H. pylori* infection. The image shows the earliest effect of mitochondria-associated changes in the relation to the different stages of gastric cancer development. Note that most of those changes are present in the later stages of this process (arrows only showing the initial impacts). Although nuclear DNA alterations are not shown in the gastric epithelial cell on this image, it is crucial in the development of gastric cancer, and it is summarized in a vignette on the right side of the image. Solid standard arrow: induction/activation; solid blunt/T-bar arrow: blocking/inhibition; dashed line: translocation. Created in BioRender. Wappler-Guzzetta, EA. (2025) https://app.biorender.com/illustrations/6933475960be5c067831d4c8?slideId=7af0b0df-ca50-421a-88a5-9c1f3fa1cd5b (accessed on 12 December 2025).

Plus, alterations in mitochondrial dynamics is a hallmark of *H. pylori* infection, characterized by increased fission and reduced fusion, and associated with decreased apoptosis [14]. Furthermore, impairment of mitophagy leads to the accumulation of damaged mitochondria, promoting oxidative stress and increase inflammatory signaling [15] (Figure 1).

### 2.1. H. pylori Infection on the Mitochondrial Functions of Gastric Epithelial Cells

*H. pylori* colonizes the mucosal surface via its virulence factors, leading to disrupted epithelial cell barrier function. The impairment of cellular integrity is primarily mediated through tight junction and adherens junction damage, which eventually results in decreased transepithelial electrical resistance (TEER), reflecting increased epithelial permeability [16]. Disruption of the epithelial barrier compromises its protective function, facilitating the translocation of luminal contents, such as acid, pepsin, and microbial products, as well as pathogens, such as *H. pylori*, into the lamina propria. These further exacerbate mucosal inflammation and injury [17]. Moreover, bacterial penetration damages the extracellular matrix in the lamina propria, which impairs epithelial cell regeneration and further disrupts tissue architecture. These contribute to chronic gastritis, and increase the risk of ulcer development and neoplastic transformation [18]. These microenvironmental alterations trigger various intracellular signaling changes, leading to mitochondrial dysfunction that predispose the cells to neoplastic transformations (Figure 1).

### 2.2. Molecular Mechanisms of Virulence Factors

*H. pylori* is a highly adapted bacterium that possesses a wide repertoire of virulence factors essential for its survival, colonization, and pathogenesis. These virulence factors include 1. urease, which neutralizes gastric acid; 2. flagella and associated motility proteins (FlaA, FlaB), which facilitate bacterial penetration through the mucus layer; 3. adhesins (such as BabA, SabA, OipA, and HopQ), which mediate attachment to the gastric epithelial cells; 4. The neutrophil-activating protein (Hp-NAP) that modulates immune responses; 5. Lipopolysaccharides (LPS), which mimic host molecules; 6. The cag pathogenicity island (cagPAI) encoding the type IV secretion system (T4SS) and its effector protein, CagA; 7. The vacuolating cytotoxin A (VacA); 8. γ-glutamyl transpeptidase (GGT); and 9. various enzymes (such as phospholipases) and antioxidants (such as catalase and superoxide dismutase) [19,20,21] (Table 1).

Among these, VacA and CagA stand out as the most critical virulence factors for inducing mitochondrial dysfunction due to their direct translocation into host cells. Virtually all *H. pylori* strains produce VacA, a secreted pore-forming toxin that permeabilizes mitochondrial membranes, whereas CagA is expressed in only ~60–70% of strains harboring the cagPAI, and is injected via T4SS. CagA has been described to modulate mitochondrial dynamics and mitophagy [22,23,24].

**Table 1:** The role of *H. pylori* virulence factors in infection and in mitochondrial damage.

Virulence Factors	Role in Host Cell Infection	Role in Mitochondrial Damage
Urease	neutralizing gastric acid [19]	-
Flagella and associated motility proteins (FlaA and FLaB)	facilitate penetration to mucus layer [19]	-
Adhesins (BabA, SaBA, OipA, HopQ)	mediate attachment to gastric epithelial cells [19]	-
neutrophil associated protein (Hp-Nap)	modulates immune responses [20]	-
Lipopolysaccharides (Lp)	mimic host molecules [19]	-
CagA (encoded by CagPAi)	Key points: interaction with the SRC homology 2 domain (SH2)-containing tyrosine phosphatase SHP-2, in activation of the RAS-ERK/MAPK pathway, modulation of actin cytoskeleton dynamics, aberrant cell morphology, cell migration, and adhesion, promotes downstream oncogenic signaling through CagA-SHP2 complexes, nuclear factor of activated T cells (NFAT) activation, and β-catenin stabilization, contributing to gastric carcinogenesis; multimerization leads to aberrant cell morphology [22] (see below for more details)	Key points: contribution to carcinogenesis by inhibiting apoptosis (see Table 6); inhibits VacA endocytosis and modifies its cellular trafficking; infection with cagPAI-positive strains alter VacA subcellular localization- potentially enhancing mitochondrial targeting, amplifying mitochondrial stress [22] (see below for more details details)
T4SS (type 4 secretion system, encoded by CagPAI)	delivers CagA to host cells [22]	-
VacA	vacuolization and internalization to host cells [23,25] (see below in details)	modulating mitochondrial dynamics, mitophagy translocation to mitochondria (TIM and TOM), dissipation of membrane potential, release of pro-apoptotic (cytochrome c), activates Bax and Bcl-2 family proteins and caspase-9/3= leads to intrinsic apoptosis VacA sustains CagA for persistence [23] (see below for more details)
Enzymes (GGT, phospholipases, catalases, superoxid dismutase)	helps surviving oxidative stress [19]	

#### 2.2.1. Vacuolating Cytotoxin A (VacA)

The vacuolating cytotoxin A (VacA) of *H. pylori* is a key factor responsible for inducing pronounced vacuolization in host cells. Upon binding to the cell surface, VacA is internalized and causes the formation of large intracellular vacuoles, which display characteristics of both late endosomes and early lysosomes. This vacuolization results primarily from VacA forming anion-selective channels in the vesicle membranes, leading to chloride ion accumulation and subsequent osmotic swelling. Although severe vacuolization is present in in vitro experiments, it is less prominent in human gastric tissue [25]. Besides its vacuole formation, VacA exerts direct effects on mitochondrial function, contributing to cellular injury and apoptosis [26].

VacA is synthesized as a ~140 kDa protoxin, proteolytically processed into p33 (N-terminal) and p55 (C-terminal) subunits, which remain non-covalently associated. The N-terminal hydrophobic domain of p33 appears critical for its mitochondrial targeting and membrane insertion. It likely allows the protein to engage with the outer mitochondrial membrane protein translocase complex (Translocase of the Outer Membrane [TOM]), and subsequently with the inner mitochondrial membrane protein translocase complex (Translocase of the Inner Membrane [TIM]), analogous to canonical presequence- or carrier-dependent import pathways [27,28,29].

The p55 domain is primarily responsible for host cell receptor binding, interacting with RPTPβ/α, lipid rafts, and sphingomyelin, and facilitating endocytosis. Previous studies suggested that the p33 subunit, but not the p55 subunit of VacA, could enter mitochondria to modulate organelle function [30]. Furthermore, crystallography studies revealed that both subunits are required for a physiologically stable pore and p55 mediates initial oligomerization between adjacent subunits. They can form flower-like hexadecameric or dodecameric assemblies upon acidification, which is essential for membrane insertion [31]. Reconstitution experiments demonstrated that independently expressed recombinant p33 and p55 can reassemble into functional oligomers, restoring vacuolating and channel-forming activities, confirming their interdependent roles [32].

VacA exhibits significant allelic polymorphism that profoundly influences its toxicity, cellular tropism, and disease association [33]. The *vacA* gene is mosaic-structured and categorized into three main variable regions: the signal sequence (s1/s2), mid-region (m1/m2), and intermediate region (i1/i2). The s1/m1 (especially s1/i1/m1) combination is recognized as the most pathogenic, strongly correlating with peptic ulceration, gastric atrophy, and adenocarcinoma risk (up to 5-fold higher than other strains) [34,35]. The s1/m1 strains produce higher levels of vacuolating activity and exhibit enhanced cellular tropism compared to s2 or m2 variants [36]. In contrast, s2 or m2 variants show reduced toxicity: the s2 allele encodes an N-terminal hydrophilic extension that impairs channel formation, secretion, and membrane insertion, while m2 strains produce a truncated toxin with limited cellular tropism and vacuolating activity [37,38]. These polymorphisms explain geographic disease variation—s1/m1 strains predominate in high-GC regions like East Asia—and synergize with cagA-positive strains to amplify mitochondrial stress and carcinogenesis [37].

Once localized to mitochondria, VacA assembles into oligomeric anion-selective channels within the inner membrane, primarily permeable to chloride ions. It was calculated that even a single channel is capable of dissipating the mitochondrial membrane potential (Δψm), which is essential for ATP synthesis and mitochondrial homeostasis. This membrane potential dissipation triggers the release of pro-apoptotic factors, such as cytochrome c, activates Bax and Bcl-2 family proteins and caspase-9/3, culminating in intrinsic apoptosis [30,39].

#### 2.2.2. Cag Pathogenicity Island (cagPAI) and Cytotoxin-Associated Gene A (CagA)

Most *H. pylori* strains contain a ~40 kb gene cluster known as the cag pathogenicity island (cag PAI). These cag PAI- containing strains show enhanced potential to induce mtROS and inflammation, correlating with increased virulence and gastric disease severity [22,40].

The cag PAI contains about 30 to 31 genes, including cagA and multiple genes essential for the assembly and function of the type IV secretion system (T4SS), which is required for delivery of CagA into host cells [20,41]. CagA comprises an N-terminal secretion domain (for T4SS recognition); three conserved arginine-rich regions (ARR1-3) that facilitate plasma membrane binding to phosphatidylserine; a central multidomain region interacting with host effectors; and a variable C-terminal region harboring 1–10 Glu-Pro-Ile-Tyr-Ala (EPIYA) motifs [22]. Once internalized, CagA is phosphorylated at its glutamate-proline-isoleucine-tyrosine-alanine (EPIYA) motifs by host kinases c-Src and c-Abl [28]. It enables its interaction with the SRC homology 2 domain (SH2)-containing tyrosine phosphatase SHP-2 [42,43]. The SH2 domain-containing inositol 5-phosphatase 2 (SHIP2, INPPL1) is a lipid phosphatase that hydrolyzes phosphatidylinositol 3,4,5-trisphosphate [PI(3,4,5)P_3_] to phosphatidylinositol 3,4-bisphosphate [PI(3,4)P_2_]. Locally increased PI(3,4)P_2_ levels activate the RAS-ERK/MAPK pathway, modulate actin cytoskeleton dynamics, cell migration, and adhesion [44]. Moreover, it leads to cytoskeletal rearrangements, increased inflammation, enhanced permeability, disrupted epithelial barrier, and it promotes uncontrolled cell proliferation.

This phosphatidylinositol alteration strengthens integrin-mediated *H. pylori*-host cell attachment (via CagL-α5β1) and stabilizes bacterial microcolonies, thereby potentiating subsequent T4SS-mediated CagA delivery [44]. By facilitating greater CagA influx, SHIP2 indirectly promotes downstream oncogenic signaling through CagA-SHP2 complexes, Nuclear Factor of Activated T cells (NFAT) activation, and β-catenin stabilization, contributing to gastric carcinogenesis. In addition, CagA directly inhibits apoptosis [45].

The key domain of CagA phosphorylation, the EPIYA motifs are classified as A, B, C, and D type [43]. While A and B are ubiquitously expressed, *H. pylori* frequently contains the C type motif in strains from Western countries, and D type motif is often seen in East Asian strains [44]. The “Western-type” CagA (EPIYA-C) binds SHIP2 more avidly than the “East Asian-type” CagA (EPIYA-D), though both subtypes engage the SH2 domain of SHIP2 following Src/Abl kinase-mediated phosphorylation [45]. The EPIYA-D motif exhibits higher binding affinity, which leads to more pronounced pathogenetic effects [29]. Interestingly, strains carrying EPIYA-D are associated with higher risk of gastric cancer development due to their enhanced SHP-2 binding affinity [46]. They also demonstrate a more prominent effect on intracellular signaling and ROS production. However, the EPIYA-C motif can also induce host gene transcription involved in gastric cancer progression, such as erbB2, HGF-R, FGFR4 and TGF-ß [47].

**Figure 2:**
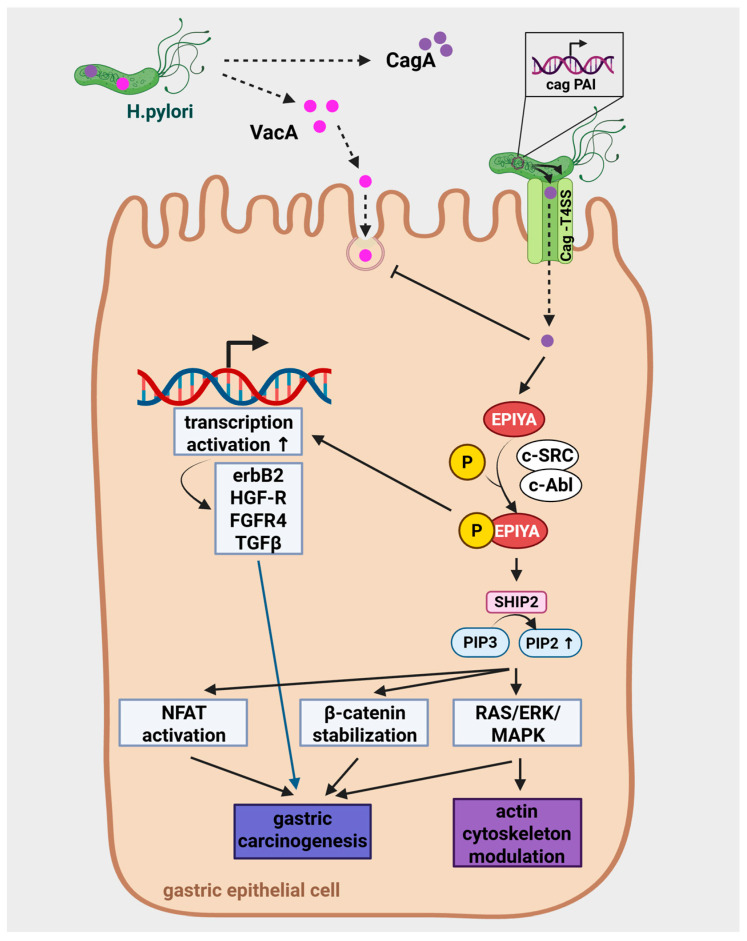
The pathogenetic pathways of *H. pylori* CagA toxin in gastric epithelial cells. Phosphorylation of the EPIYA motifs leads to SHIP2 activation, which increases local PIP2 level. This activates signal transduction pathways, leading to gastric carcinogenesis and actin cytoskeleton modulation. Created in BioRender. Wappler-Guzzetta, EA. (2025) https://app.biorender.com/illustrations/6965087a2f45a20605d8a70a?slideId=7af0b0df-ca50-421a-88a5-9c1f3fa1cd5b (accessed on 30 January 2026).

Additionally, CagA effect can be enhanced by its multimerization: multimerization motifs within the C-terminal region of the CagA protein, known as CagA-multimerization (CM) motifs or CM-like sequences, are critical for its ability to self-associate (oligomerize) within the host cells. These motifs consist of approximately 16 amino acid residues located immediately downstream of the last EPIYA phosphorylation segment. The CM motifs enable CagA to form dimers or higher-order oligomers independently of its phosphorylation status, which markedly enhances its biological activity [22,48]. Structurally, CM motifs serve as interaction interfaces facilitating head-to-tail binding between CagA molecules, thereby stabilizing multimeric complexes. This multimerization significantly increases the strength of CagA’s interactions with host signaling proteins, especially the SH2 domain-containing phosphatase SHP2, a critical mediator of CagA-driven oncogenic signaling pathways [48,49]. CM motif repeats exponentially increase CagA binding affinity for SHP2, which amplifies its downstream effects, such as aberrant cell morphology (the “hummingbird phenotype”, which is the dramatic actin-dependent elongation of gastric epithelial cells), disrupted cell polarity, and enhanced proliferative signaling [48,49] (Figure 2). The hummingbird phenotype is linked to increased cellular motility and scattering, crucial for *H. pylori*-induced pathogenesis [50].

#### 2.2.3. Interactions Between VacA and CagA

The interaction between VacA and CagA consists of multilevel functional antagonistic and synergistic effects. This “yin-yang” regulation sustains chronic, non-lethal inflammation. VacA can clear ”competitors”, as it is a multifunctional toxin that creates membrane channels, induces vacuoles, and impairs autophagy. Also, it helps *H. pylori* to survive by suppressing T-cell activation and proliferation, disabling the host’s immune response against the bacteria [51]. While CagA drives transformation (via SHP2/β-catenin pathway), VacA sustains CagA for persistence.

On the other hand, CagA expression can inhibit VacA endocytosis and modify its cellular trafficking [52]. Additionally, co-infection with cagPAI-positive strains alters VacA subcellular localization, potentially enhancing its mitochondrial targeting and efficiency through T4SS-mediated crosstalk or shared receptor utilization, thereby amplifying mitochondrial stress [26,53].

Interestingly, CagA mediated PI3K/Akt activation and NFAT nuclear translocation counteract VacA-induced changes of mitochondrial membrane potential, cytochrome c release, and apoptosis. Also, CagA and VacA have shown to inhibit each other’s effects on epithelial cells in the morphological level: CagA reduces VacA -associated vacuolation, whereas VacA reduces hummingbird formation [50]. On the other hand, CagA-induced mitophagy clears VacA-damaged mitochondria, and balances ROS to prevent apoptosis while sustaining chronic stress. Plus, VacA can stabilize CagA levels by inhibiting CagA degradation. In VacA negative *H. pylori* strains, therefore, host mechanisms degrade 70% of injected CagA within 6 h.

Further studies on VacA–CagA interaction showed that VacA’s lipid raft disruption impairs lysosomal targeting of CagA. Moreover, VacA reduces glutathione, sensitizing cells to H_2_O_2_, which is a byproduct of CagA induced SMOX activity [54]. They also share NF-κB signaling pathways, where CagA induces canonical (p65/RelA), and VacA induces the non-canonical (RelB/p52) pathways, counterbalancing inflammation [55] (also see Table 2). CagA+-only strains have predominantly pro-inflammatory properties, causing excessive activation of the canonical p65/RelA pathway. This leads to cell cycle arrest and robust inflammation, potentially hindering the long-term survival of the bacteria. Conversely, VacA+ -only strains have pro-apoptotic properties via the NF-κB non-canonical pathway activation [56], which leads to increased pro-inflammatory cytokine secretion (such as IL-1β, TNF-α, IL-6, and IL-8), increased level of pro-apoptotic proteins (such as Bax) and decreased levels of anti-apoptotic proteins (such as Bcl-xl) [57]. Infection with a CagA+VacA+ (high virulence) strain induces a balanced level of total NF-κB activity, promoting cell proliferation and survival, allowing for a persistent *H. pylori* infection and long-term colonization. Furthermore, the amplified toxicity by the CagA+/s1m1 VacA+ (most virulent VacA) strains are associated with higher gastric cancer/MALT lymphoma prevalence [58], (Table 2).

The coordinated mitochondrial targeting by VacA and CagA underscores *H. pylori*’s sophisticated manipulation of host cell metabolism, fostering persistence and disease progression (see Figure 1 and Figure 2). In Table 3, we summarize the most important points of interaction between VacA and CagA on the mitochondria.

#### 2.2.4. The Role of VacA and CagA in ROS/RNS-Induced Oxidative Cascade and *H. pylori*- Induced Carcinogenesis

**Figure 3:**
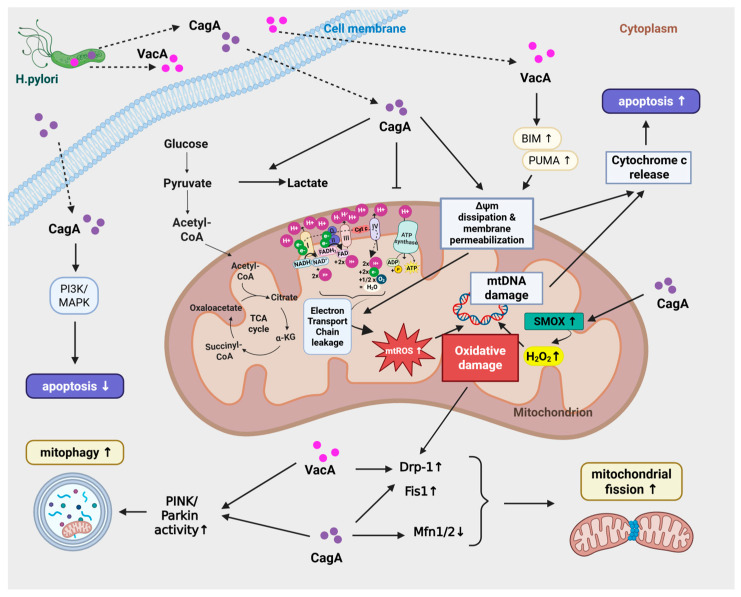
*H. pylori* induces oxidative stress via its main virulence factors, VacA and CagA. Mitochondrial ROS (mtROS)-induced oxidative damage plays a central role in *H. pylori*-associated gastric cell damage, leading to chronic inflammation and/or carcinogenesis. Created in BioRender. Wappler-Guzzetta, EA. (2025) https://app.biorender.com/illustrations/6965086ddea12a99b008f915?slideId=7af0b0df-ca50-421a-88a5-9c1f3fa1cd5b (accessed on 30 January 2026).

In addition to the previously mentioned antagonistic effect between VacA and CagA, these two virulence factors also have synergistic effects in the host cell between each other, such as the increased ROS production and subsequent oxidative damage. ROS generation is associated with the dysregulation a numerous signaling pathways, such as PI3K/AKT/mTOR, JAK/STAT3, NF-κB/MAPK, MAP/ERK/JNK, and the NRLP3 inflammasome pathway, which adds to the complexity of it downstream effects, resulting in the picture seen with the oxidative damage [61] (See Figure 3 for further details).

The effect VacA and CagA on mitochondrial ROS is well described. The channel formation of VacA directly precipitates mitochondrial ROS (mtROS) overproduction through electron transport chain (ETC) leakage and impaired glutathione metabolism following ΔΨm dissipation [61,66,67]. Furthermore, VacA initiates ROS-induced ROS release (RIRR), a propagating mechanism wherein mtROS from one mitochondrion diffuses to adjacent organelles, eliciting synchronized Ca^2+^ waves and amplified ROS bursts that facilitate inter-mitochondrial communication [58].

In contrast, CagA prominently induces the expression and activity of spermine oxidase (SMOX), a key enzyme involved in polyamine metabolism that catalyzes the conversion of spermine into spermidine, producing hydrogen peroxide (H_2_O_2_) as a reactive oxygen species byproduct [68]. Studies have demonstrated that the generation of H_2_O_2_ contributes to oxidative DNA damage, mucosal inflammation, and drives gastric epithelial cell apoptosis, thus playing a pivotal role in the pathogenesis of *H. pylori*-associated gastric cancer. A pilot study also confirmed the increased level of SMOX expression in gastric cancer patients with the history of *H. pylori* infection [69]. Genetic deletion or pharmacological inhibition of SMOX in animal models significantly reduces inflammation, oxidative DNA damage, and tumorigenic signaling, including suppression of β-catenin pathway activation, a key driver of gastric oncogenesis [70]. CagA also promotes iNOS expression in epithelial/immune cells via the NF-κB/STAT3 pathway, generating NO- that forms peroxynitrite (ONOO^−^) with superoxide [71]. This chronic oxidative/nitrosative stress overwhelms the antioxidant defense systems (e.g., SOD2, glutathione, arginase) [67,72], causing mtDNA damage, lipid peroxidation, protein carbonylation, and nitrotyrosine formation, leading to carcinogenesis [73].

#### 2.2.5. mtROS as Novel and Emerging Therapeutic Target Against *H. pylori*

*H. pylori*-induced mitochondrial ROS formation is a central player in epithelial injury, inflammation, and cancer progression, making mtROS a compelling therapeutic target. In vitro and animal studies support the usefulness of antioxidant strategies that preserve host mitochondrial function, alongside ROS-amplifying nanodrugs or small molecules that selectively overwhelm bacterial redox systems. These approaches are promising, but there is no clinical data on its safety and efficacy in clinical studies for these diseases. In Table 4, we summarize cell protective antioxidants targeting mitochondrial ROS.

The success of *H. pylori* eradication strategies continuously decline due to increased antibiotic resistance in the past decade. Several ROS-centered, largely antibiotic-free strategies are emerging for *H. pylori*, especially nanogenerators and nanozymes, which either boost bactericidal ROS locally or dampen harmful host oxidative stress. Reviews on mitochondrial ROS in infection and on mitochondria-targeted ROS nanomedicine in cancer highlight two translatable ideas: (1) to amplify ROS within pathogen or tumor mitochondria to kill the target cell, and (2) to protect or reprogram host mitochondria by modulating mtROS and antioxidant defenses [79,80,81].

Current *H. pylori* nanoplatforms primarily focus on localized extracellular/intra-bacterial ROS. A future direction is to incorporate explicit mitochondrial-targeting motifs (e.g., TPP ligands) to fine-tune mtROS in host cells, or to exploit bacteria-like energy machinery, building on cancer nanomedicine designs. In sum, ROS-centered nanogenerators (sonodynamic/chemodynamic, cascade nanozymes, MOFs) and redox-cycling antibiotics are leading emerging strategies, aiming to target multidrug-resistant *H. pylori* strains in the future. Their transition to human use will, however, require further studies [79,80,81] (See more details in Table 5).

### 2.3. H. pylori Induces Mitochondrial Dynamics Imbalance

*H. pylori* infection significantly disrupts mitochondrial dynamics, contributing to gastric epithelial cell damage and pathogenesis. Presumably, both VacA and CagA toxins can mediate mitochondrial fragmentation by recruiting dynamin-related protein 1 (Drp1), a key mediator of mitochondrial fission, to the mitochondrial surface. Fission protein 1 (FIS1) is also necessary for mitochondrial fission as it serves as binding sites for Drp1 recruitment. CagA can increase the expression of both Drp1 and FIS1 in a human gastric cancer (AGS) cell line [63]. Mitofusins (MFN1 and MFN2) are GTPases, located in the outer mitochondrial membrane, and they are essential for mitochondrial fusion. They facilitate the formation of connections between adjacent mitochondria, while the optic atrophy 1 (OPA1) protein fuses the inner mitochondrial membrane. Fusion is necessary for maintaining the integrity of the mitochondrial network, for mixing mitochondrial contents (e.g., mtDNA and proteins), and for the functional complementation of damaged organelles. CagA was shown to decrease the expression of both MFN1 and MFN2, resulting in decreased fusion in gastric epithelial cells [63]. This fragmentation disrupts mitochondrial dynamics, compromising mitochondrial network integrity and leads to decreased respiratory capacity and ATP production. Additionally, mitochondrial fission/fusion imbalance causes accumulation of damaged mitochondria, triggering mitophagy that is a quality control mechanism to remove dysfunctional organelles [63]. Inhibition of Drp1 activity in VacA-exposed cells prevents activation of the pro-apoptotic protein Bax, mitochondrial outer membrane permeabilization (MOMP), and subsequent cell death [64]. Moreover, VacA-induced mitochondrial damage induces metabolic stress pathways, including the AMPK pathway, which interplay with mitochondrial fission and autophagy to regulate toxin clearance and cell viability [53,63]. These coordinated alterations in mitochondrial dynamics and quality control mechanisms help the bacterium to maintain a favorable environment for chronic infection while driving pathogenesis through mitochondrial dysfunction [63] (See more details in Figure 1).

### 2.4. Autophagy–Mitophagy

*H. pylori* infection triggers both autophagy and mitophagy as crucial host defense mechanisms to remove damaged mitochondria and dysfunctional cellular components. VacA is a key inducer of both autophagy and mitophagy, promoting mitochondrial depolarization and stabilizing PINK1 on the outer mitochondrial membrane. PINK1 subsequently recruits Parkin for ubiquitination and LC3-mediated mitophagy [86,87]. On the other hand, VacA impairs this quality control by sustaining mitochondrial membrane potential (Δψm) perturbations, which eventually overwhelms the host cell’s mitophagic capacity, triggering apoptosis [27,63].

In contrast to VacA, CagA has a complex regulatory role in mitochondrial quality control and inflammation during *H. pylori* infection. It promotes selective mitophagy via the PINK1/Parkin pathway, which leads to the removal of damaged mitochondria. This action attenuates excessive inflammation by preventing the over-activation of the NLRP3 inflammasome, thereby balancing inflammatory responses and helping the bacterium to evade host immune clearance. By maintaining a controlled level of mtROS, CagA supports chronic infection and increases the survival and viability of the infected gastric epithelial cells [63,66].

Besides, CagA activates NADPH oxidase (NOX) through ERK/NF-κB signaling, which enhances ROS production and promotes inflammation [88], while simultaneously inhibiting general autophagy through pathways such as c-Met-PI3K/Akt/mTOR pathway, promoting cell stress [66,87] (Figure 1).

Chronic infection also suppresses autophagy via downregulation of key regulators like SIRT1 and RUNX3, contributing to impaired cellular clearance and promoting inflammation [27,88].

Overall, the interplay between *H. pylori* virulence factors and host autophagy pathways represents a dynamic balance where autophagy/mitophagy attempts to mitigate mitochondrial damage and control inflammation, but *H. pylori* can influence these pathways in order to survive, however it may also lead to gastric disease progression.

### 2.5. Apoptosis

*H. pylori* promotes gastric carcinogenesis through a complex balance between epithelial apoptosis and survival, heavily modulated by its virulence factors VacA and CagA. Their interaction determines whether cells die, survive with DNA damage, or enter a hyperproliferative state, favoring cancer.

In cases when mitophagy is insufficient to remove damaged mitochondria, both intrinsic and extrinsic apoptotic pathways can be activated. Additionally, VacA plays a major role in the initiation of apoptosis, while CagA can modulate, and in some extend counteract its effect.

VacA primarily activates the intrinsic (mitochondrial) apoptotic pathway by generating ROS, and causing subsequent DNA damage. This upregulates BH3-only proteins such as Bim and PUMA, activating BAX and BAK, which, together with VacA, permeabilize the mitochondrial outer membrane, dissipate Δψm, and trigger cytochrome c release. This cytochrome c release leads to caspase-9 and caspase-3 activation, causing apoptosis [89,90]. VacA can also intersect with the non-canonical forms of programmed cell death. In some gastric cell models, VacA-induced mitochondrial dysfunction, ER stress, and ATP depletion drive a mixed phenotype cell death, including apoptosis and autophagic cell death [91], or programmed necrosis [92,93].

As previously mentioned, CagA has an opposite role in apoptosis induction. VacA activates PI3K signaling pathways that dampens apoptosis, required for *H. pylori*-induced cell proliferation [59]. This PI3K-driven pro-survival signaling cooperates with MAPK pathways. In gastric cancer cells, inhibition of any of the three major MAPK branches—ERK (via MEK1/2), p38, or JNK—enhances apoptosis following *H. pylori* infection, indicating that MAPKs normally provide partial protection from cell death during infection [94]. Consistent with this, ERK1/2 and p38 activation is more pronounced in cagA+ strains and that this activation protects against epithelial apoptosis at least in part by upregulating the anti-apoptotic protein Bcl-2 [95]. Beyond kinase pathways, CagA can directly interfere with apoptotic control by reducing the expression of tumor-suppressive E3 ubiquitin ligases such as SIVA1 and ULF in SNU1 gastric cancer cells, a mechanism proposed to support gastric tumor development [96]. Additionally, CagA can also protect epithelial cells from VacA-induced mitochondrial apoptosis by preventing VacA trafficking to the mitochondria [52]. These findings support a model in which VacA drives early mitochondrial apoptosis and tissue damage, whereas CagA affects apoptotic regulators, limiting excessive cell loss, and promoting a chronic, pro-carcinogenic infection. In Table 5, we summarized the interplay between vacA and cagA in apoptosis regulation and their role in the development in gastric cancer and MALT lymphoma: VacA both antagonizes CagA phenotypes (through vacuolation vs. hummingbird morphology) and promotes CagA accumulation via autophagy disruption, potentially amplifying CagA oncogenic signaling. Meanwhile, CagA limits VacA entry and blocks VacA-induced apoptosis, helping to maintain long-term colonization and chronic inflammation, favoring carcinogenesis. Interestingly, while different VacA alleles carry a different risk of carcinogenesis. Furthermore, amino acid polymorphisms of both VacA and CagA have also been associated with GC or MALT lymphoma development [97] (see Table 6).

**Table 6:** The role of VacA and CagA in apoptosis and carcinogenesis (gastric cancer and MALT lymphoma).

Aspect	VacA	CagA
Direct effect on epithelial cell apoptosis	Strongly pro-apoptotic; activates the intrinsic (mitochondrial) apoptotic pathway by generating ROS and DNA damage, upregulating BH3-only proteins such as Bim and PUMA, activating BAX and BAK, which, together with VacA, permeabilize the mitochondrial outer membrane, dissipate Δψm, and trigger cytochrome c release, leading to caspase-9 then caspase-3 activation and ultimately to apoptosis [96,97] induces mitochondrial and caspase-8/9-dependent apoptosis, G1 arrest, and autophagic cell death in gastric epithelial/cancer cells	Predominantly anti-apoptotic in gastric epithelium; CagA blocks VacA-induced apoptosis at trafficking and mitochondrial levels; lead to the activation of PI3K signaling pathways that dampens apoptosis and is required for *H. pylori*-induced cell migration [26]. PI3K-driven pro-survival signaling cooperates with MAPK pathways [94]. ERK1/2 and p38 activation in cagA^+^ strains protects against epithelial apoptosis at least in part by upregulating the anti-apoptotic protein Bcl-2 [95]. CagA reducing the expression of tumor-suppressive E3 ubiquitin ligases such as SIVA1 and ULF in SNU1 gastric cancer cells (interferes with apoptotic control), a mechanism proposed to support gastric tumor development
Net effect on gastric epithelial homeostasis	Increases apoptosis and cell death; may trigger compensatory hyperproliferation, metaplasia and mucosal atrophy, contributing indirectly to carcinogenesis [93]	Drives proliferation and survival, dissociating proliferation from apoptosis in cagA+/vacA s1a infections, a pattern linked to higher gastric cancer risk [57]
Role in gastric cancer (epidemiologic/experimental)	Highly toxigenic vacA s1/i1/m1 alleles associate with intestinal metaplasia and gastric cancer; vacA i1 strongly linked to precancerous metaplasia in humans and to severe metaplasia/inflammation in mice [98,99]. VacA contributes to chronic inflammatory damage via TRAF1–4-1BB–NF-κB–IL-8 axis [57]	CagA is a major oncoprotein. In a carcinogenic gerbil strain, cagA deletion abolishes gastric cancer, whereas vacA deletion does not, pinpointing CagA as essential for adenocarcinoma development 19. CagA+ strains correlate with enhanced epithelial cell proliferation and cancer risk [100,101]
VacA–CagA functional interplay in gastric cancer	VacA both antagonizes CagA phenotypes (through vacuolization vs. hummingbird morphology) and promotes CagA accumulation via autophagy disruption, potentially amplifying CagA oncogenic signaling [51,52]	CagA limits VacA entry and blocks VacA-induced apoptosis, helping maintain long-term colonization and chronic inflammation that favor carcinogenesis [52,101]
Association with gastric MALT lymphoma risk	Systematic review shows no clear pro-MALT role; vacA genotypes show an inverse or non-significant association with MALT lymphoma risk, likely because VacA-induced apoptosis does not favor B-cell tumorigenesis [102]	Meta-analysis: no significant association between cagA status and classic gastric MALT lymphoma, but strong association with diffuse large B-cell lymphoma (DLBCL) (OR ~6.4) where CagA in B cells activates ERK, p38, BCL2, NF-κB, and inhibits p53/JAK-STAT to promote tumorigenesis [102]
Sequence differences in GC vs. MALT strains	Specific VacA amino acid polymorphisms differ between gastric cancer and MALT strains, despite similar conventional genotypes; suggests subtle VacA structural variants may bias toward GC vs. MALT, though function not yet defined [97]	Distinct CagA amino acid polymorphisms are found in different frequencies in GC vs. MALT strains; one CagA site also differs between gastritis and MALT isolates, consistent with disease-specific CagA variants affecting B-cell vs. epithelial cell tropism and signaling [97]

Other virulence factors can also influence the apoptotic pathways. For example, gamma-glutamyl transpeptidase (GGT) inhibits apoptosis and induces gastric epithelial cell proliferation through the induction of cyclooxygenase-2, epidermal growth factor-related peptides, and interleukin-8 [103].

*H. pylori* can also activate the extrinsic, death-receptor-mediated apoptotic pathway, which is tightly regulated and is often counterbalanced, especially during chronic infection. In gastric epithelial cells, infection upregulates Fas and sensitizes cells to TNF-related apoptosis-inducing ligand (TRAIL) and TNF-α, promoting engagement of death receptors such as Fas, TRAIL-R1/R2, and TNFR1. This receptor ligation leads to assembly of the death-inducing signaling complex (DISC), recruitment of FADD and procaspase-8, promote caspase-8 activation, and cleavage of BID, which links the extrinsic pathway to mitochondrial permeabilization and intrinsic apoptosis [104,105]. Furthermore, *H. pylori* can suppress apoptosis by inducing factors, such as FRA-1 [106], and by differentially affecting cell in the acute vs. chronic settings. While acute exposure accelerates epithelial apoptosis, prolonged infection reduces *H. pylori*-induced apoptosis, helping infected cells to survive [107,108]. Collectively, these data support the view that an imbalance between apoptosis and proliferation-induced by *H. pylori* virulence factors contributes to gastric carcinogenesis, and that eradication of *H. pylori* can at least partially normalize these processes [109].

### 2.6. mtDNA Mutations

*H. pylori* infection promotes genomic instability in gastric epithelial cells, affecting both the nuclear and the mtDNA. While the mutagenic impact on nuclear DNA partly stems from suppressed expression and activity of key DNA repair pathways, *H. pylori* similarly impairs mtDNA integrity, leading to increased mutation frequency, reduced mtDNA copy number, and compromised respiratory function [110]. In gastric adenocarcinoma cell lines (e.g., AGS cells), *H. pylori* exposure triggers mtDNA mutations predominantly in the hypervariable D-loop region—a non-coding regulatory element critical for mtDNA replication and transcription—along with rare heteroplasmic point mutations in protein-coding genes like cytochrome b (Cytb). The primary driver of *H. pylori*-induced mtDNA mutations is oxidative stress from mtROS overproduction, fueled by virulence factors, such as VacA. This oxidative burden causes base lesions, single and double-strand breaks, adducts formation and base mismatch and depletion of mtDNA content, as observed in both in vitro models and gastritis patient biopsies [67,111].

Mitochondrial base excision repair (mtBER) is a primary mechanism for correcting oxidative mtDNA damage with its key enzyme called apurinic/apyrimidinic endonuclease 1 (APE-1). Y-box binding protein 1 (YB-1) is implicated in mismatch repair, while DNA polymerase gamma (POLG), and DNA polymerase beta (POLB) would fill DNA gaps [13]. DNA ligase 3 is also a key element of the BER pathway, both in the nucleus and in the mitochondria. It interacts with tyrosyl-DNA phosphodiesterase 1 (TDP1), with NEIL1/2 glycosylases, and with POLG [112]. During *H. pylori* infection, oxidative stress leads to accumulation of mtDNA lesions, which triggers activation of BER pathways. YB-1 has been identified as participating in mitochondrial DNA repair, cooperating with APE-1 to maintain mitochondrial genome integrity under infection-induced stress [113]. Experimental knockdown of mitochondrial APE-1 and YB-1, heightens *H. pylori*-induced mtDNA mutation loads. These findings confirm that APE-1 and YB-1 actively protect mtDNA during infection, with multiple repair pathways converging to mitigate oxidative damage. However, persistent infection overwhelms these repair systems, leading to mitochondrial dysfunction and increased susceptibility to carcinogenesis [113].

The mitochondrial protein import complex, translocase of outer membrane (TOM), plays a crucial role in importing nuclear-encoded proteins required for mtDNA maintenance [28]. During early stages of infection, VacA transiently enhances mitochondrial import machinery and key factors for mtDNA replication and transcription such as POLG and TFAM; at later stages, however, VacA’s effect on these processes diminishes [13].

MtDNA damage can lead to leakage of mtDNA fragments into the cytoplasm, caused by mitochondrial dysfunction and oxidative stress. Once in the cytoplasm, mtDNA acts as a damage-associated molecular pattern (DAMP), and it is sensed by the cytoplasmic cyclic GMP-AMP synthase (cGAS), which produces cyclic GMP-AMP (cGAMP). cGAMP then activates stimulator of interferon genes (STING), triggering a downstream signaling cascade that induces type I interferon production and pro-inflammatory cytokines, integral to antiviral and inflammatory responses. Persistent activation of this cGAS-STING pathway by mtDNA from damaged mitochondria sustains chronic inflammation, contributing to gastric pathology associated with *H. pylori* infection [114].

Mitochondria has other ways to maintain quality control and mtDNA integrity, such as by mitochondrial fusion, which allows the compensation of damaged mtDNA with normal mtDNA (maintaining the mutation rate below 80%) [115], or by enabling the removal of damaged mtDNA with mitophagy [116]. As previously mentioned, both options are highly affected during *H. pylori* infection, leading to mutagenesis.

Furthermore, previous studies have investigated the association between mtDNA copy number in peripheral blood and GC risk. Some studies showed that higher mtDNA copy number is a risk factor to develop GC [117] or associated with poor prognosis with reduced survival period [118]. However, a recent article with an extensive meta-analysis found no discernible causal relationship between peripheral blood mtDNA copy number and GC [119].

In conclusion, *H. pylori*-mediated oxidative stress induces mtDNA damage that disrupts mitochondrial function. During early infection it promotes the increase of mitochondrial translocases and some mtDNA repair factors, such as POLG. However, during later stages of the disease, the repair mechanisms are overwhelmed, and DNA mutations are accumulating. In the nucleus, well-known oncogenes are induced, while in the mitochondria, genetic instability activates innate immune responses through the cGAS-STING pathway, promoting chronic inflammation and carcinogenesis in the gastric mucosa.

### 2.7. Mitochondrial Bioenergetics During H. pylori Infection

*H. pylori* infection induces profound alterations in mitochondrial bioenergetics, which contribute to the metabolic reprogramming underlying gastric pathogenesis and carcinogenesis. As previously mentioned, VacA toxin targets mitochondria and impairs their function by disrupting the mitochondrial membrane potential, decreasing ATP production, and inducing metabolic stress within infected gastric epithelial cells. This mitochondrial dysfunction caused by VacA leads to reduced oxidative phosphorylation efficiency and triggers compensatory metabolic shifts towards glycolysis, a hallmark of cancer metabolism known as the “Warburg effect” [120].

Despite initial mitochondrial damage, host cells activate adaptive mechanisms for mitochondrial restoration. Sensing cellular energy deficits via AMP-activated protein kinase (AMPK) leads to enhanced mitochondrial fission and turnover, which facilitates clearance of mitochondrial toxins and helps recover mitochondrial function and ATP levels in a time-dependent manner. Mitochondrial fission and autophagy work coordinately to maintain metabolic homeostasis even under continued VacA exposure [121].

Additionally, ATP-dependent Lon protease, a mitochondrial matrix protease crucial for degradation of damaged proteins and maintenance of mitochondrial proteostasis, is upregulated during *H. pylori* infection. The Lon protease protects mitochondrial function by eliminating proteins that are damaged by oxidative stress, preserving respiratory chain integrity and preventing excessive ROS production. However, persistent infection and prolonged stress overwhelm this protease system, resulting in progressive mitochondrial dysfunction and increased susceptibility to malignant transformation [122].

Collectively, these studies demonstrate that *H. pylori*-induced mitochondrial disruption leads to impaired oxidative phosphorylation and energy production, rerouting cellular metabolism towards glycolysis. At the same time, mitochondrial quality control mechanisms such as AMPK-mediated fission and Lon protease activation attempt to restore bioenergetic balance. Failure of these compensatory responses contributes to mitochondrial dysfunction, oxidative stress, and gastric carcinogenesis, highlighting mitochondrial bioenergetics as a critical axis in *H. pylori* pathobiology and a potential therapeutic target.

### 2.8. H. pylori-Induced Dysregulation of Immune Cells

*H. pylori* exerts a variety of effects on different immune cell types through its virulence factors and metabolic products, modulating both innate and adaptive immunity to promote persistent infection.

The bacterium induces production of cytokines and chemokines from both gastric epithelial cells and from immune cells [123,124,125]. The multiple cytokines in the gastric mucosa (including TNF, IFN-γ, IL-1β, IL-6, IL-8, and IL-18) are predicted to have pro-inflammatory effects, whereas IL-10 and TGF-β are cytokines that may limit the inflammatory response. The impaired integrity and basal membrane damage thus create a microenvironment favorable for persistent infection, chronic inflammation, and carcinogenesis. It is partially caused by mtDNA mutations and impaired repair mechanisms, leading to genomic instability and prolonged mitochondrial dysfunction [13]. Specifically, *H. pylori* activates NF-κB and MAPKs pathways in gastric epithelial cells, leading to secretion of IL-8, IL-6, IL-1β, and TNF-α. Members of the innate immune system (monocytes/macrophages and dendritic cells) will produce high levels of IL-1β, IL-6, TNF-α, IL-10 and IL-12 upon *H. pylori* infection. Kranzer et al. showed that human dendritic cells exposed to *H. pylori* produce IL-6, IL-8, IL-10, IL-12, and TNFa and they undergo maturation, however, DC activation and maturation are independent of the cagPAI and VacA status of *H. pylori* [126] (Figure 1).

Dendritic cells (DCs) also have the capacity to induce effector T cells at the mucosal sites. Khamri et al. demonstrated that *H. pylori*-stimulated dendritic cells can promote IL-1β/IL-23-dependent IL-17 production by CD4^+^ T cells [126]. D4+ T cells, also known as Th cells are divided into Th1, Th2, Th17, Th22, Th9, regulatory T cells (Tregs), and follicular helper T cells (Tfh). *H. pylori* exert a complex CD4^+^ T-cell response involving Th1, Th17, and regulatory T cells (Treg), each defined by characteristic cytokines. *H. pylori* stimulate dendritic cells to produce IL-12 to promote the differentiation to the Th1 type. Th1 cells mainly produce interferon-γ (IFN-γ), IL-2, and IL-12. IFN-γ can activate macrophages and strengthen their ability to phagocytose and kill *H. pylori*. Th17 cells differentiate from naïve CD4+ T cells in the presence of cytokines such as TGF-β, IL-6, IL-21 and IL-23. Th17 cells secrete IL-17, IL-21, IL-22 and IL-23, which recruit and activate neutrophils, induce epithelial antimicrobial peptides, chemokines, and matrix-metalloproteinases, and contribute to chronic inflammatory damage [127].To limit excessive immune response and permit bacterial persistence, *H. pylori* expands regulatory T cells in the gastric mucosa. TGF-β plays a key role in the differentiation of induced Tregs. These Treg cells typically produce high levels of IL-10 and TGF-β, which suppress Th1/Th17 effector functions, dampen local inflammation, and impair bacterial clearance [128,129]. *H. pylori* in general inhibits T-cell proliferation and activation, partly through the action of VacA which interferes with calcium signaling and mitochondrial function in T cells, leading to cell cycle arrest or apoptosis. Additionally, *H. pylori* skews T helper cell differentiation towards Th1 and Th17 and Treg, which are balancing the level of inflammation while fail an effective bacterial clearance [128,130].

IFN-γ produced by Th1 or gastric cells cannot only induce phagocytosis by the macrophages, but also to produce bactericidal substances such as nitric oxide and reactive oxygen species, enhancing inflammation [131]. One of the host’s defense strategies against *H. pylori* involves macrophage production of nitric oxide (NO) via inducible nitric oxide synthase (iNOS). In vitro studies have demonstrated that macrophages co-cultured with *H. pylori* can restrict bacterial growth through NO-dependent mechanisms. However, this bactericidal activity is often insufficient because the availability of the iNOS substrate L-arginine is limited. *H. pylori* induces arginase activity in macrophages, which competes for and depletes L-arginine, thereby reducing NO synthesis and enabling bacterial survival [131,132].

In macrophages, SMOX activity reduces intracellular spermine levels, an inhibitor of inducible nitric oxide synthase (iNOS), thereby promoting iNOS protein expression and nitric oxide (NO) production that enhances antimicrobial defense against *H. pylori*. Conversely, inhibition or knockdown of SMOX worsens bacterial clearance by impairing NO-mediated killing [72]. However, elevated NO can contribute to the buildup of reactive nitrogen species (RNS). Together with another byproduct of SMOX catalyzed reaction-H_2_O_2_ can enhance inflammatory pathways, mitochondrial dysfunction and mtROS production. Furthermore, SMOX-generated 3-aminopropanaldehyde is converted into the highly reactive electrophile acrolein. In gastric epithelial cells of H.p infected mouse models, acrolein forms stable adducts with DNA and proteins, causing genotoxic stress, DNA strand breaks, and mutations. Pharmacological scavengers of acrolein, such as 2-hydroxybenzylamine (2-HOBA), mitigate DNA damage and suppress gastric carcinogenesis in experimental models, highlighting the therapeutic potential of targeting SMOX-acrolein axis [68].

Neutrophils belong to one of the earliest and most abundant immune cells recruited upon *H. pylori* infection to the gastric mucosa. It is navigated by several factors, including the neutrophil-activating protein NapA, urease, and chemokines (notably IL-8) [133]. These factors can mediate the release l-selectin (CD62L) expressed on the cellular surface, with a subsequent upregulation of the β2-integrins CD11b and CD11c, which are essential for transendothelial migration of neutrophils to areas of inflammation [134].Once neutrophils are activated they will be the major source of reactive oxygen species (ROS), myeloperoxidase, and pro-inflammatory cytokines including IL-8, IL-1β, TNF-α, and IL-10, thereby amplifying local inflammation and recruiting additional leukocytes [135]. This persistent ROS release and sustained inflammation contributes to epithelial injury, DNA damage, and, over the long term, gastric carcinogenesis.

Chronic inflammation with elevated cytokine level drives persistent mtROS production triggering activation of the NLRP3 inflammasome complex. The activation mechanism involves mtROS facilitating the release of mitochondrial damage-associated molecular patterns (DAMPs), including oxidized mitochondrial DNA, which promote NLRP3 inflammasome assembly. This leads to caspase-1 activation, processing, and release of pro-inflammatory cytokines IL-1β and IL-18, amplifying gastric mucosal inflammation [20,63]. MtROS also directly activates NLRP3 by thioredoxin-interacting protein (TXNIP) dissociation from thioredoxin, an event that triggers inflammasome assembly independent of potassium (K^+^) efflux. However, *H. pylori* limits full caspase-1 activation, enabling bacterial persistence despite inflammasome priming [136,137]. Early during infection (around 6 h), *H. pylori* upregulates NLRP3 mRNA expression via activities of cag pathogenicity island (cagPAI) and VacA toxins, but by 24 h, NLRP3 protein levels are suppressed through induction of microRNA miR-223-3p and anti-inflammatory cytokine IL-10. This regulation results in accumulation of pro-IL-1β but limits production of the mature cytokine IL-1β, modulating the inflammasome output and preventing excessive inflammatory damage. However, external stimulation by microbial or environmental activators readily induce NLRP3 inflammasome formation and secretion of high amounts of mature IL-1beta cytokines in humans [136]. This fine-tuned control allows mitochondrial stress to prime the inflammasome without triggering full activation and pyroptotic cell death, thereby maintaining a state of chronic gastritis conducive to bacterial persistence and sustained host inflammation [138].

*H. pylori* infection could facilitate the persistence of follicles on which continuous follicular helper T-cell activation could lead to uncontrolled follicular B-cell proliferation. Gastric mucosa B cells (which are antigen-presenting cells; APCs) internalize bacterial antigens (for example urease, flagellin, outer membrane proteins), presenting them to T helper cells. Upon activation, antigen-specific T helper cells will express CD40L which interact with CD40 on the B cells and drives their entry into S phase; simultaneously, Th1 cytokines (IL-2, IFN-γ) and Th2 cytokines (IL-4, IL-5, IL-6, IL-10, IL-13) promote clonal expansion, antibody secretion, and isotype switching from IgM to IgG, ultimately leading to the differentiation into plasma cells and memory B cells [139]. B cells produce antibodies, predominantly IgA at the mucosal surface and IgG systemically, which can neutralize bacterial factors and promote clearance, although this humoral response alone rarely eradicates the infection. 

Chronic stimulation by *H. pylori* also drives expansion of regulatory B cells (Bregs), typically IL-10, producing CD24^+^CD38^+^ subsets, which dampen Th1/Th17 responses and contribute to bacterial persistence by suppressing excessive inflammation. Over years, sustained B-cell activation within acquired gastric mucosa-associated lymphoid tissue creates a setting in which genetic lesions such as *BCR3–MALT1* and other translocations can arise, leading to monoclonal proliferation and gastric MALT lymphoma [140].

*H. pylori* also inhibit the STING and RIG-I signaling via downregulation of IRF3 activation, suppressing type I interferon responses [141]. This immune modulation reduces bacterial clearance while promoting a tolerogenic environment facilitating chronic infection.

In summary, *H. pylori* orchestrates a multifaceted disruption involving epithelial and immune cells, modulating acid secretion, promoting mitochondrial ROS generation, and sustaining low level of chronic inflammation. The immune-modulatory strategy that simultaneously activates inflammatory responses and impairs immune clearance, contributing to persistent infection and inflammation-associated gastric malignant transformation.

## 3. The Role of Mitochondrial Stress in *H. pylori*-Linked Gastric Cancer

### 3.1. Different Haplotypes Polymorphism and Strains of H. pylori and Their Pathogenic Features

Although around 50% of people are infected with *H. pylori*, only a very small percentage will develop cancer. The likelihood of developing malignancies reflects an interaction between bacterial strain diversity, host genetic polymorphisms, and population background. Certain *H. pylori* haplogroups, such as the East Asian strains carry highly virulent constellations of virulence factors. On the host side, functional polymorphisms in the genes of pro- and anti-inflammatory cytokines (for example IL-1ß, TNFα, IL-10) and innate immune receptors such as TLR4 can modify the inflammatory response to the infection. Combinations of high-risk host genotypes and high-risk bacterial strains can amplify gastric cancer odds up to ten-fold relative to low-risk constellations.

A meta-analysis using data from BioBank Japan found that Germline pathogenic variants in nine genes (APC, ATM, BRCA1, BRCA2, CDH1, MLH1, MSH2, MSH6, and PALB2) were associated with the risk of gastric cancer [142].

*H. pylori* has many strains, which exert very different virulence and malignant potential. CagA is widely recognized as the first identified bacterial oncoprotein, classified as a class 1 oncoprotein by the WHO, and CagA-positive strains elevate GC odds ratios 2-5-fold. The risk of GC is further increased when individuals are infected by strains expressing the CagA containing a high number of repeats its C’-terminal variable region or the D type of this EPIYA motif [143]. VacA s1/m1 alleles synergize by exacerbating epithelial damage. Infection induces hypochlorhydria via parietal cell loss, promoting bacterial overgrowth and carcinogenic nitrite formation.

Additionally, CM motif polymorphisms—variations in sequence and copy number—differentiate Western-type and East Asian-type CagA. Western strains typically harbor two CM motifs with moderate SHP2-binding affinity, while East Asian CagA possesses a single but higher-affinity CM motif variant, correlating with increased virulence and gastric cancer risk in East Asian populations [48].

Overall, the CM motif-mediated oligomerization of CagA serves as a molecular amplifier, potentiating CagA-SHP2 interactions and oncogenic signaling cascades critical for *H. pylori* pathogenesis.

### 3.2. Gastric Cancer-Specific Mitochondrial Alterations Beyond Acute Infection

*H. pylori*-induced mitochondrial stress in GC manifests as a shift toward the Warburg effect, with upregulated glycolysis and suppressed oxidative phosphorylation (OXPHOS) supporting proliferation under hypoxia. Complex I deficiency from mitochondrial DNA (mtDNA) mutations reduces ATP production, downregulating peroxisome proliferator-activated receptor gamma coactivator 1-alpha (PGC1α) while upregulating glutaminase (GLS1)-driven glutaminolysis. Phosphorylated (Ser616) Drp1 hyperactivation coupled with Mfn1 and OPA1 suppression generates fragmented mitochondria that enhance invasion and metastasis [113].

Mitophagy is first induced in order to remove damaged mitochondria. CagA is helping this process, which favors pro-survival signaling without triggering apoptosis. However, later the damage will overwhelm the clearance, and damaged mitochondria will be accumulated. The impaired PTEN-induced kinase 1/Parkin (PINK1/Parkin)/microtubule-associated protein 1A/1B-light chain 3 (LC3-II) favors apoptosis resistance through Bcl-2 upregulation/Bax downregulation. Transcription factor A, mitochondrial (TFAM) downregulation and DNA polymerase gamma (POLG) upregulation fail to compensate for mtDNA loss, perpetuating genomic instability characteristic of GC progression [113].

### 3.3. mtROS-Mediated Apoptosis Evasion and Tumorigenesis

*H. pylori* infection-associated mtROS play a complex role in gastric cancer, contributing both to apoptotic cell death and to tumorigenesis by facilitating apoptosis evasion. Elevated mtROS levels can induce mitochondrial membrane depolarization, cytochrome c release, and activation of caspase-dependent apoptosis, serving as a tumor-suppressive mechanism to eliminate damaged cells. Several anticancer drugs exploit this by enhancing mtROS production to trigger apoptosis and cell cycle arrest in gastric cancer cell lines [144].

Conversely, chronic mtROS generation can promote gastric tumor progression by activating oncogenic signaling pathways such as β-catenin/Wnt, MAPK, and STAT3, and by inducing DNA mutations and genomic instability [141]. Tumor cells often develop mechanisms to evade mtROS-induced apoptosis, including upregulation of antioxidant defenses and alteration of mitochondrial dynamics, allowing them to tolerate oxidative stress and sustain proliferation.

Thus, mtROS create a dual effect in gastric cancer biology: low to moderate ROS levels act as signaling molecules promoting tumor growth and survival, while excessive ROS trigger apoptosis.

The mtROS-induced mtDNA mutations [113] accumulate through the Correa cascade and correlate with Lauren histotypes; intestinal-type GC shows mtDNA stability, while diffuse-type exhibits hypermutation. The mtROS-NF-κB loop sustains STAT3/YAP/β-catenin signaling, promoting cadherin 1 (CDH1) loss—a diffuse GC hallmark—and peritoneal metastasis.

### 3.4. Mitochondria-Targeted Therapeutic Strategies for H. pylori-Associated GC

*H. pylori* eradication remains the cornerstone (30–50% GC risk reduction), though antibiotic resistance limits efficacy. Chemotherapy is the major treatment for gastric cancer especially in advanced cancer stages, however the combination of two or even three agents are preferable due to their synergism and lower side effects. Targeted therapy in gastric cancer is currently directed towards cell surface and signaling molecules (HER2, VEGF/VEGFR, PD-1/PD-L1, CLDN18.2, FGFR, etc.) and not towards mitochondria. However, mitochondrial metabolism and mitochondrial stress responses are increasingly recognized as key determinants of response and resistance to these agents and to chemotherapy in general [145,146].

In the treatment of gastric and gastro-oesophageal cancers, HER2-directed antibodies (trastuzumab, trastuzumab deruxtecan), VEGFR2 blockade (ramucirumab), PD-1 inhibitors (pembrolizumab, nivolumab), and other biomarker-guided agents in trials (FGFR2, CLDN18.2, MET, EGFR) are mainly used. These remain largely non-mitochondrial targets [147,148].

Mitochondria are central to energy, redox control, apoptosis, and oncometabolite production; stress-adaptive changes in these functions support survival under therapy-induced stress. Resistant tumor cells often shift toward high OXPHOS and enhanced mitochondrial stress responses (UPRmt, integrated stress response, chaperones such as HSP90/TRAP1), which buffer oxidative and proteotoxic stress, and promote chemoresistance [149,150].

Targeting these adaptations (OXPHOS inhibitors, mtDNA replication inhibitors, mitochondrial HSP90/TRAP1 inhibitors, metformin, CPI-613, “mitocan” chemotherapeutics) is under active investigation to resensitize tumors to standard therapies [151,152]. Across standard drugs, small-molecule inhibitors, phytochemicals, nanomedicines, and mitochondrial transplantation, a common theme is pushing mitochondrial ROS and dysfunction beyond the adaptive capacity of gastric cancer cells, often to overcome chemoresistance or cancer stemness, while exploiting cancer-specific redox vulnerabilities: nanoparticle-delivered mitochondrial antioxidants overcome resistance, potentiating cisplatin efficacy. Clinical trials of using Drp1 inhibitors after *H. pylori* eradication also show potential therapeutic benefit [113,153]. In Table 7, we summarize mitochondrial stress and mtROS related aspects of gastric cancer treatment: how key, currently studied or emerging gastric cancer therapies intersect with mitochondrial stress/ROS.

## 4. Mitochondrial Stress in *Helicobacter pylori*-Associated MALT Lymphoma

### 4.1. Helicobacter pylori Infection in MALT Lymphoma: Pathogenic Significance

The prevalence of *H. pylori* infection is very high in patients diagnosed with gastric MALT lymphoma, highlighting the role of *H. pylori* infection in its pathogenesis [168]. The unique ability of *H. pylori* to manipulate host immune responses, including the downregulation of T-cell cytotoxicity and the induction of tolerogenic dendritic cell phenotypes, supports the persistence of lymphoma.

Unlike gastric cancer (cagA^+^ vacA s1/m1 dominant), MALT lymphoma associates with less virulent *H. pylori* strains, optimized for chronic immune evasion [169].

The pathogenesis of MALT lymphoma involves a longer antigenic stimulation without the apoptosis of the host cells. The CagA negative are dominantly the responsible strains for the pathogenicity. The genotyping of the strain can predict eradication success; cagA negative, without t(11;18) translocation are the easiest to cure with rituximab treatment.

This longer immunological stimulation induces lymphoid follicles in the gastric mucosa, then evolve to the polyclonal lymphoid hyperplasia and further toward the generation of oligoclonal then monoclonal B-cell population [6]. The reasons behind this progression, however, are not fully understood.

The progress of the disease can be linked directly and indirectly to *H. pylori* infection. The indirect route is through *H. pylori*- specific T helper cells connecting to the B cells via CD40L/CD40, triggering various downstream intracellular pathways. Additionally, FOXP3+ regulatory T-cells release cytokines, chemokines, and costimulatory molecules participate in the evolution and maintenance of the neoplastic B-cell population [170]. On the other hand, CagA can directly phosphorylate SHP-2, and activate the downstream signaling, either through ERK- p38MAPK, or via Bcl-XL/Bcl-2 to induce proliferation and to inhibit apoptosis.

**Figure 4:**
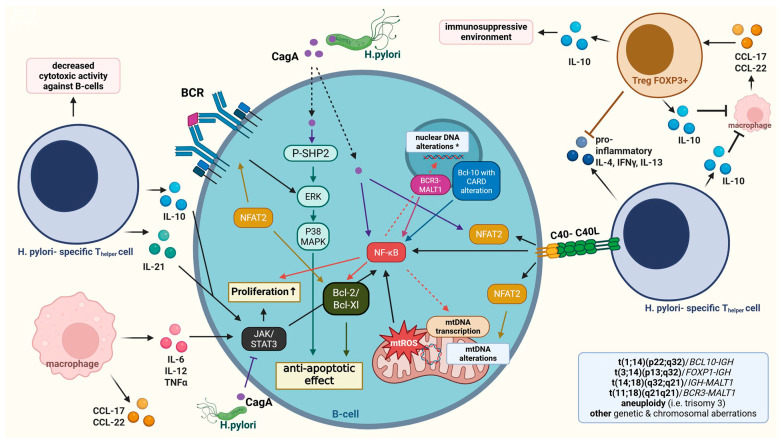
In gastric MALT lymphoma, chronic *Helicobacter pylori*-driven signaling integrates *H. pylori* virulence factors with B-cell receptor (BCR) and T-cell-dependent pathways to promote antigen-driven proliferation and cell survival, and later antigen-independent tumor cell proliferation. CagA, delivered by the bacterial type IV secretion system, can engage SH2-domain-containing host proteins, contributing to the activation of ERK and p38 MAPK. This reinforces a pro-survival, pro-proliferative environment that favors the expansion of antigen-stimulated B cells. Our knowledge of the role of mitochondria in MALT lymphoma development and progression is limited, with mitochondrial reactive oxygen species (mtROS) production, mtDNA alterations, and NF-κB effect on mtDNA transcription being the main involvement according to current literature. NF-κB, a central molecule in MALT-lymphoma development and progression, can translocate to the mitochondria, binding to the mtDNA promoter regions to upregulate TFAM and CYTB, sustaining OXPHOS and mtROS-dependent survival signaling. In parallel, persistent cytokine and receptor signaling converges on NF-κB, which becomes constitutively active once characteristic chromosomal translocations arise, such as *BCR3*–*MALT1* or *BCL10/IGH* rearrangements, locking in survival and growth signals even in the absence of ongoing antigen presence. Within the lymphoma clone, chronic BCR engagement and CD40 ligation by *H. pylori*-specific T-helper cells are central drivers of altered intracellular signaling. BCR downstream signaling provides Ca^2+^ flux and mitochondrial ROS that leads to sustained NFAT2 activation, while NF-κB both cooperates with NFAT2 on target genes and can upregulate Nfatc1 itself. This NFAT2–NF-κB–mitochondria axis helps in the maintenance of cell survival, ongoing proliferation, and resistance to apoptosis in the marginal-zone B-cell clone, creating a permissive background in which NF-κB-activating chromosomal translocations (such as *BCR3–MALT1/BCL10*) can induce antigen-independent cell proliferation and cell growth. Furthermore, both NF- κB and NFAT2 upregulate the expression of anti-apoptotic molecules, such as Bcl-2/Bcl-Xl, further shifting the cell survival balance towards an anti-apoptotic state. In addition, the complex cytokine signals present in the *H. pylori*- infected gastric mucosa further promotes B-cell survival, proliferation, and apoptosis inhibition. These cytokines include the pro-inflammatory IL-4, IFNγ, IL-13, IL-6, IL-12, TNFα, CCL-17, and CCL22; and the anti-inflammatory IL-10 and TGFβ. The constellation of inflammatory cells and their cytokine expression pattern results in an immunosuppressive environment and decreased CD4+ cell cytotoxic effect in the *H. pylori*-infected gastric mucosa, fostering the development of MALT lymphoma. Over time, the combination of sustained NF-κB signaling and *H. pylori*-conditioned cytokine milieu facilitates the selection of B-cell clones harboring NF-κB-activating chromosomal translocations, completing the transition from reactive lymphoid hyperplasia to antigen-independent MALT lymphoma. The most common genetic alterations are listed in the vignette in the right bottom part of the image. Solid standard arrow: induction/activation; Solid blunt/T-bar arrow: blocking/inhibition; Dashed line: translocation. Created in BioRender. Wappler-Guzzetta, EA. (2025) https://app.biorender.com/illustrations/6939ad8b8e22a2d07bb5d84e?slideId=8a60beb1-e4f2-42f8-a428-920db0d7648d (accessed on 12 December 2025).

At the molecular level, chronic stimulation favors the acquisition of characteristic chromosomal translocations such as t(11;18)(q21;q21) *BCR3*–*MALT1*, t(1;14)(p22;q32) *BCL10*-*IGH*, and t(14;18)(q32;q21) *IGH*-*MALT1*. *BCR3*-*MALT1* translocation (t(11;18)) in 20–30% confers *H. pylori*-independent signaling through the canonical NF-κB pathway [171]. By contrast, the t(1;14)(p22;q32) translocation drives overexpression of BCL10, often with alterations in its CARD (caspase recruitment domain), which likewise leads to constitutive NF-κB activation. Collectively, these genetic lesions have established aberrant activation of the classical NF-κB pathway, which is a central mechanism in the pathogenesis of MALT lymphoma (Figure 4).

### 4.2. Mitochondrial Stress in MALT Lymphoma

Emerging evidence positions NF-kB signaling as a key player in MALT lymphoma pathogenesis. In early, *H. pylori*-dependent MALT lymphoma, NF-κB activation is primarily extrinsically driven by chronic BCR and CD40 signaling, with modulation by CagA-induced ERK/p38 MAPK pathways and cooperation from NFAT2. This stage remains reversible with *H. pylori* eradication. However, the continuous antigen stimulation leads to chromosomal translocations, such as t(11;18)(q21;q21), t(1;14)(p22;q32), and t(14;18)(q32;q21). In this stage NF-κB becomes intrinsically and constitutively active, locking in B-cell survival and proliferation regardless of ongoing infection. NF-kB then exert many anti-apoptotic effects and promote proliferation; for example, upregulates genes such as Bcl-2, Bcl-Xl, and various inhibitors of apoptosis, and also induces cell cycle regulators. The latter mechanism drives antigen-independent proliferation, explaining why these lymphomas often fail to regress after *H. pylori* eradication. In addition, NF-κB upregulates chemokines and cytokines that attract and polarize T cells and other immune cells, helping maintain the CD40L- and cytokine-rich niche that further stimulates BCR/CD40 and NF-κB itself. NF-κB activation can even enhance the expression of *BCR3–MALT1* fusion gene, creating a self-reinforcing loop. This NF-κB–centered signaling architecture is the core molecular engine that drives the transition from reactive lymphoid hyperplasia to overt, *H. pylori*-independent MALT lymphoma [172].

In the case of the gastric epithelial cells, VacA-induced mitochondrial depolarization and outer membrane permeabilization can trigger cytochrome c release and apoptosis. Therefore, we can hypothesize a similar mechanism in B cells, where chronic inflammation can lead to a persistent, sublethal level of mtROS production. We assume that mtROS can further enhance NF-kB activation via three mechanisms: One, mtROS generated at complexes I and III can oxidatively activate redox-sensitive kinases such as c-Src and MAP3Ks, which lie upstream of the IKK complex. Activated IKK phosphorylates IκBα, leading to its degradation and the release of NF-κB (typically p65/p50) to translocate into the nucleus and drive transcription of target genes. Two, ROS promote tyrosine phosphorylation (rather than the classic serine phosphorylation and degradation) of IκBα, which can also permit NF-κB activation without complete IκBα proteolysis. This mechanism has been shown in hypoxia and TNF-α models. Three, NF-κB induces expression of mitochondrial antioxidants like MnSOD (SOD2) and other redox regulators. This would create a feedback loop where mtROS activate NF-κB, and NF-κB then partially restrains mtROS to keep them at a “signaling” rather than a “toxic” level [173].

It is important to mention that while low level mtROS typically enhance NF-κB activity and favor survival, inflammation, and pro-survival gene expression, high mtROS or oxidative burst would inhibit IKK or damage NF-κB components, or push the cell toward apoptosis instead of adaptive signaling. In case of B cells, the low-grade mtROS output from stressed mitochondria is entirely consistent with sustained NF-κB activation and survival signaling, even though the exact quantitative relationship in MALT has not been tested experimentally.

Evidence from biochemical fractionation and imaging shows that NF-κB subunits such as RelA and IκBα can be found in mitochondrial fractions and can bind mitochondrial DNA regulatory regions, where they modulate transcription of some mitochondrial genes (for example COX subunits and Cyt b) [172]. Import of IκBα into mitochondria is stimulus-dependent (e.g., TNFα, hypoxia) and may involve components of the outer-membrane import machinery such as TOM40 [146]. During early hypoxia, studies showed that a RelA/IκBα complex transiently accumulates in mitochondria and it was dependent on STAT3 phosphorylation. Cooperation between STAT3 and the RelA/IκBα complex has been described to preserve mitochondrial function and cell viability [174,175].

Mitochondrial RelA/IκBα has been implicated in fine-tuning OXPHOS, mtROS production, and apoptosis sensitivity by adjusting expression of selected mtDNA-encoded respiratory chain components. Therefore mitochondrial NF-κB signaling helps tumor cells to survive by downregulating apoptosis and regulating mitochondrial stress (Figure 2).

### 4.3. Mitochondrial Stress-Related Aspects of MALT Lymphoma Therapy

In MALT lymphoma, the current treatment guideline is based on *H. pylori* eradication therapy, and conventional lymphoma modalities. No mitochondrial or mtROS-targeted drugs are used. Oxidative stress and ROS, however, are important in the pathogenesis of MALT lymphoma, with mitochondria-targeted therapies remaining experimental concepts without current routine application in this disease [176].

## 5. Conclusions and Future Perspectives

Besides its common and widely known pathological and microbiological effects, *H. pylori* infection is responsible for mitochondrial stress-driven GC and MALT lymphoma tumorigenesis and cancer progression. Our current knowledge in this field, however, is limited. Future preclinical and clinical studies investigating the role of *H. pylori*- associated effects on mitochondrial stress, mtROS, mitochondrial metabolism, and mitochondrial dynamics may lead to new therapeutic targets in these malignancies.

In *H. pylori* infection, therefore in prevention of neoplasia, gastric-localized ROS nanogenerators that eradicate *H. pylori* (especially multiresistant strains for overcoming the widespread problem if antibiotic resistance) while sparing microbiota and limiting mucosal mtROS injury can be a promising future direction in developing new drugs. Further discovery of long-term mtROS-modulating nutraceuticals (similar compounds like astaxanthin, ginseng, Nrf2 activators) as chemopreventive adjuncts, if possible, treatment optimized by monitoring biomarkers of oxidative stress can prevent *H. pylori* caused oxidative damage on host cells.

In gastric cancer, mitochondria-targeted pro-oxidant nanomedicines can potentially selectively trigger mtROS-driven apoptosis and overcome chemoresistance. Furthermore, combination of mtROS-modulating agents with immunotherapy could reprogram the immunosuppressive tumor microenvironment, enhancing antitumor immunity. Precision targeting of redox defenses (Prx2/Prx3, NOX, antioxidant enzymes) and mtROS downstream pathways, such as JAK2/STAT3 or TLR4–Akt–NF-κB, are further future anticancer targets.

Although there is no mtROS data regarding MALT lymphoma; extrapolation from *H. pylori*-driven ROS biology suggests to explore mtROS-sparing eradication regimens and redox-modulating agents to prevent MALT lymphoma progression.

The mtROS sits at the nexus of inflammation, cancerous transformation, and therapy response in *H. pylori* infection and gastric cancer. Promising future treatments therefore include mitochondria-targeted pro-oxidant nanoplatforms, and combinations of mtROS modulators with immunotherapy or eradication regimens in gastric cancer. In MALT lymphoma, translation of these concepts remains an important research gap.

## Figures and Tables

**Table 2:** Interactions between CagA and VacA, and their role in carcinogenesis [56]. *: level of activation. ↑: increased expression. ↓: decreased expression.

NFκB Signaling	*H. pylori* Strain			Effect
	CagA+/VacA+	CagA Only	VacA Only	
canonical p65/RelA	moderate *	high *	low *	proliferation
non-canonical RelB/p52	moderate *	low *	high *	pro-inflammatory (IL-1β, TNF-α, IL-6, IL-8 ↑) and pro-apoptotic effect (Bax ↑, Bcl-xl ↓)
Total NFκB activity	balanced *	excessive (cell cycle arrest)	ineffective (apoptosis)	persistent infection, long- term colonization

**Table 3:** The most important points of interaction between VacA and CagA on mitochondrial level.

Level of Interaction	VacA	CagA	Association with Carcinogenesis
Common Goal: maintenance of chronic non-lethal inflammation			
Internalization to host cell, subcellular localization	CagA inhibits VacA endocytosis & modifies its cellular trafficking; infection with cagPAI-positive strains alter VacA subcellular localization [59]		-
Effect on mitochondrial membrane potential	VacA dissipates mitochondrial membrane potential, therefore induces cytochrome c release and apoptosis; CagA mediated PI+K/Akt activation and NFAT nuclear translocation counteract these effects [50,52].		-
Stabilization/clearance	VacA inhibits CagA degradation [26]	CagA induces mitophagy, clears VacA-infected mitochondria	-
NFκB pathways	induces non-canonical pathways	induces canonical pathways	see Table 1
ROS-mediated oxidative stress	directly precipitates mitochondrial ROS (mtROS) production through electron transport chain (ETC) leakage, and impairs glutathione metabolism following ΔΨm dissipation	induces the expression and activity of spermine oxidase (SMOX), a key enzyme involved in polyamine metabolism that catalyzes the conversion of spermine into spermidine, producing hydrogen peroxide (H_2_O_2_) [54] promotes iNOS expression in epithelial/immune cells via NF-κB/STAT3, generating NO- that forms peroxynitrite (ONOO^−^) with superoxide	- 8-OHdG, 8-nitroguanine accumulation, double-strand mtDNA breaks [60] - Activation of NF-κB, Akt, MAPK pathways [61] - Mitochondrial dysfunction, glycolytic shift, more ROS (self-perpetuating oxidative stress loop) [62]
Mitochondrial dynamics	inhibiting Drp1 activity [63]	recruiting Drp1, increases Drp1 and Fis1 expression, decreases MFN1 and MFN2 expression [63]	- VacA directly targets mitochondria, recruits Drp1, induces mitochondrial fission, cytochrome c release, and apoptosis in epithelial cells [64]
Mitophagy-autophagy	promotes mitophagy and autophagy: stabilization of PINK1, recruiting Parkin for ubiquitination and mitophagy; impairs quality control by sustaining mitochondrial membrane potential (Δψm) perturbations, overwhelming mitophagic capacity; promoting apoptosis [27]	promotes mitophagy via the PINK1/Parkin pathway, which leads to the removal of damaged mitochondria; balancing inflammatory responses and helping *H. pylori* to evade host immune clearance; maintaining a controlled level of mitochondrial ROS (mtROS); supports chronic infection and increases the survival and viability of infected gastric epithelial cells; cagA activates NADPH oxidase (NOX) through ERK/NF-κB signaling, which enhances ROS production and promotes inflammation	- CagA induces mitochondrial oxidative damage, dysfunction, and dynamic imbalance, and triggers mitophagy while blocking autophagy; inhibiting mitophagy increases NLRP3 activation and apoptosis, reducing survival of infected cells [63] - CagA also interacts with CYP11A1, driving mitochondrial cholesterol accumulation, inhibiting mitophagy, maintaining mitochondrial homeostasis, and promoting gastric cancer cell proliferation and apoptosis resistance [65]
Apoptosis	VacA activates the intrinsic (mitochondrial) apoptotic pathway by generating ROS and DNA damage, upregulating BH3-only proteins (Bim and PUMA9), activating BAX and BAX → mitochondrial outer membrane permeabilization → triggers cytochrome c release → caspase-9/caspase-3 activation → apoptosis.	CagA activates PI3K signaling pathways that cooperates with MAPK. In gastric cancer cells, inhibition of any of the three major MAPK branches—ERK (via MEK1/2), p38, or JNK—enhances apoptosis, ERK1/2 and p38 activation is more pronounced in cagA+ strains → upregulating Bcl-2 → protects against epithelial apoptosis.	see Table 6.

**Table 4:** Host protective strategies targeting mtROS by using antioxidants. AA2G: ascorbyl glucoside. ↑: increased expression.

Agent/Approach	Main Effect in Preclinical *H. pylori* Models
Astaxanthin	It lowers mitochondrial & total ROS, restores membrane potential & ATP, reduces NF-κB and IL-8 via PPAR-γ/catalase activation [74]
Korean red ginseng	It activates Nrf2 → ↑SOD-1, HO-1; reduces ROS, IL-8, and mitochondrial dysfunction [75]
N-acetylcysteine	It lowers ROS, oxidative DNA damage, and PI3K/Akt activation in vitro and in mice [76]
AA2G (stable vitamin C), vitamin E	They decrease oxidative stress, improve mitochondrial function, and reduce apoptosis [77,78]

**Table 5:** Comparison of nanoparticle-based treatment strategies against *H. pylori* infection. Fe-HMME@DHA@MPN: pH-responsive reactive oxygen species (ROS) nanogenerator composed of acid-responsive metal polyphenol network (MPN) shell and mesoporous metal-organic nanostructure core [Fe-HMME (hematoporphyrin monomethyl ether, sonosensitizer)] loaded with dihydroartemisinin (DHA); PtCo@G@H2A: dual-targeted cascade catalytic nanozyme PtCo@Graphene@Hemin-2(L-arginine); Bi-MOF@CS-Se: selenized chitosan (CS-Se) modified bismuth-based metal-organic framework (Bi-MOF@CS-Se) nanodrug; ICG@FCS: chitosan-conjugated fucose loaded with indocyanine green sonodynamic nanoplatform.

Strategy/Platform	Main Mechanism (ROS/Mitochondria Related)	Key Anti-*H. pylori* Features	Effect on Microbiota/Host
Fe-HMME@DHA@MPN gastric-acid-responsive ROS nanogenerator	Sonosensitizer core + DHA; in gastric acid, Fe^2+^ drives Fenton/Fenton-like reactions to generate O_2_, •OH and singlet oxygen; strong local ROS burst [82]	Kills multidrug-resistant *H. pylori*, removes biofilms, high efficacy in a mouse model [82]	Negligible disruption of intestinal flora vs. triple therapy [82]
PtCo@G@H2A dual-targeted cascade nanozyme	pH-responsive oxidase-like activity generates ROS, which further oxidizes L-arginine to bactericidal NO; acid-selective [83]	Dual targeting (hemin receptor + charge reversal) yields ~8.5-fold higher targeting; potent killing in gastric acid/pepsin [83]	Surface charge reverses in intestinal fluid to avoid off-target effects [83]
Bi-MOF@CS-Se mucoadhesive nanodrug	Bi-MOF releases Bi^3+^ in gastric acid; CS-Se layer modulates oxidative stress and inflammatory ROS in host tissue [84]	Strong activity against standard and antibiotic-resistant *H. pylori*; mucin adhesion improves gastric retention [84]	Alleviates inflammation and excessive ROS, preserves gut microbiota homeostasis [84]
Sonodynamic ICG@FCS nanoplatform	Ultrasound-activated ICG produces singlet oxygen (ROS); promotes autophagy to clear intracellular bacteria [85]	Dual targeting via fucose and ultrasound; eradicates planktonic, biofilm, and intracellular *H. pylori*, including MDR strains [85]	Minimal effect on gut microbiota; supports gastric mucosal repair [85]

**Table 7:** Mitochondrial stress and mitochondrial ROS-related aspects of gastric cancer treatment.

Therapy/Strategy	Therapeutic Class/Target	Mitochondrial Stress/ROS Mechanism	Stage/Context
5-FU-based chemotherapy (±others)	Standard cytotoxic	Increases ROS; efficacy and resistance modulated by mitochondrial metabolism and redox buffering [154,155,156,157]	Standard of care; basis for many combinations
Ruxolitinib + chemotherapy	JAK1/2 inhibitor, chemosensitizer	Reduces mitochondrial respiration, increases ROS, triggers mitochondrial apoptosis, overcomes chemoresistance [158]	Preclinical GC cell and xenograft models
CYT997	Microtubule-targeting agent	Induces mitochondrial ROS accumulation; mitoROS inhibits JAK2/STAT3, causing G2/M arrest, autophagy and apoptosis [156]	Preclinical; PDX models in GC
DS18561882 (MTHFD2 inhibitor)	One-carbon/redox metabolism inhibitor	Lowers NADPH/GSH, impairs mitochondrial function, raises ROS, promotes cell death under hypoxia [159]	Preclinical; cell lines and PDX models
Resveratrol	Natural polyphenol	Disrupts mitochondrial USP36–SOD2 axis, causes mitochondrial damage and ROS accumulation, induces autophagy-ferroptosis [160]	Preclinical; GC cells and xenografts
Phytochemicals targeting mitochondria (berberine, daidzein, tetrandrine, celastrol, shikonin, etc.)	Bioactive natural products	Promote cytochrome c release, caspase activation; increase ROS and decrease mitochondrial membrane potential to drive apoptosis [141,161]	Preclinical; proposed for drug development
ROS-modulating nanomedicine (e.g., HMON@CuS/Gd=CuS-modified hollow mesoporous organosilica nanoparticles)	Photothermal/photodynamic nanoplatform	NIR (near infrared induced photothermal effect)-triggered ROS damages mitochondrial membrane potential, boosts mtROS, opens mPTP (mitochondrial permeability transition pore), releases cytochrome c, activates caspase-9/3 [162]	Preclinical GC models
General ROS-dependent nanocarriers	ROS-responsive nano-delivery systems	Use tumor ROS or amplify ROS (often mitochondrial) for controlled release and tumor killing [154,163]	Preclinical; design frameworks
Mitochondrial transplantation + 5-FU	Cell therapy/metabolic reprogramming	Transplant normal gastric mitochondria → increase oxidative stress and apoptosis, enhance 5-FU chemosensitivity, reduce stemness [164]	Preclinical; GC cells and xenografts
PINK1-defect-based combination (Mdivi-1 + indomethacin)	Mitochondrial dynamics/mitophagy-targeted combination	Impairs Drp1-mediated fission in PINK1-low cells, causing mitochondrial pathology-mediated cell death while sparing normal cells [165]	Preclinical; GC cell models
Targeting antioxidant/redox regulators (NRF2, MTHFD2, Prx/Trx systems)	Redox-signaling–directed therapies	Disrupt redox homeostasis so mitochondrial and cellular ROS exceed survival threshold, sensitizing to therapy [154,155,159,166,167]	Conceptual & preclinical

## Data Availability

No new data were created or analyzed in this study. Data sharing is not applicable to this article.

## Abbreviations

Abbreviations are detailed in the text at their first appearance and listed here:

8-OHdG	8-hydroxy-2′-deoxyguanosine
AGS	human gastric cancer cell line
Akt	Ak strain transforming protein kinase B
AMPK	AMP-activated protein kinase
APC	adenomatosus polyposis coli gene/protein
APE-1	Apurinic/apyrimidinic endonuclease 1
APE-1	apurinic/apyrimidinic endonuclease 1(APE-1)
ARR 1-3	conserved arginin rich regions og CagPAI
ATM	Ataxia-Telangiectasia Mutated
ATP	adenosin triphosphate
BabA	Blood group antigen-binding adhesin
BAK	Bcl-2 antagonist/killer
Bax	Bcl-2-associated X protein
Bcl2	B-cell lymphoma 2 gene/protein
Bcl-xl	B-cell lymphoma-extra large
BCR	B-cell receptor
BH3-only proteins	
BID	BH3 interacting-domain death agonist
Bim	Bcl-2 interacting mediator of cell death
Bi-MOF@CS-Se	selenized chitosan (CS-Se) modified bismuth-based metal-organic framework nanodrug
BRCA1	breast cancer gene 1
BRCA2	breast cancer gene 2
Breg	regulatory B cells
CagA	cytotoxin-associated gene A
CagL-α5β1	integrin responsible for adhesion of CAgA
cagPAI	Cag pathogenicity island
CARD	caspase reccruitment domain
CD11b, CD11c	β2 integrin
CD40/CD40L	Cluster of differentiation 40 surface antigen/ligand
CD62L	release 1 selectin
CDH1	cadherin 1
cGAS	cyclic GMP-AMP synthase
cGAS-STING pathway	cyclic GMP-AMP synthase-stimulator of interferon genes- pathway
cGMP	cyclic GMP
CLDN18.2	claudiin 18 isoform 2
CM motifs	CagA-multimerization motifs, CM-like sequences
COX	cyclooxygenase
CPI-613	experimental developmental code for devimistat
CYP11A1	Cytochrome P450 Family 11 Subfamily A Member 1
CYT997	lexibulin dihydrochloride
CytB	cytochrome B
DAMP	damage-associated molecular pattern
DISC	death-inducing signaling complex
DNA	deoxyribonucleic acid
Drp1	dynamin-related protein 1
DS18561882	MTHFD2 inhibitor
EGFR	epidermal growth factor receptor
EPIYA	glutamate-proline-isoleucine-tyrosine-alanin motif
ER	endoplasmic reticulum
ERBB2	erythroblastic oncogene B tyrosin kinase B
ERK	extracellular signal-regulated kinase
FADD	Fas associated death domain
Fas	FS-7 associated surface antigen
FGFR	fibroblast growth factor receptor
FGFR2	fibroblast growth factor receptor 2
FGFR4	fibroblast growth factor receptor 4
Fis1	mitochondrial fission protein 1
FlaA	flagella associated motility protein A
FlaB	flagella associated motility protein B
FRA-1	Fos-related antigen 1
GGT	γ-glutamil transpeptidase
GSH	reduced glutathion
*H. pylori*	*Helicobacter pylori*
HER2	human epidermal growth factor receptor (similar to ErbB2)
HGF-R	hepatocyte growth factor
HMME@DHA@MPN	pH-responsive reactive oxygen species (ROS) nanogenerator composed of acid-responsive metal polyphenol network (MPN) shell and mesoporous metal-organic nanostructure core [Fe-HMME (hematoporphyrin monomethyl ether, sonosensitizer)] loaded with dihydroartemisinin (DHA)
HMON@CUS/Gd=CuS	modified hollow mesoporous organosilica nanoparticles
HopQ	Helicobacter outer membrane protein Q
Hp-NAP	*H. pylori* neutrophil-activating protein
HSP90/TRAP1	heat shock protein 90/Tumor Necrosis Factor Receptor-Associated Protein 1
ICG@FCS	chitosan-conjugated fucose loaded with indocyanine green sonodynamic nanoplatform
IFNγ	interferon γ
IgG	immunglobuline G
IgM	immunglobuline M
IKK	Inhibitor of kappaB kinase
IL 10	interleukin 10
IL 12	interleukin 12
IL 17	interleukin 17
IL 18	interleukin 18
IL 1β	interleukin 1β
IL 23	interleuki 23
IL 4	interleukin 4
IL 5	interleukin 5
IL 8	interleukin 8
IL-1β	interleukin 1β
iNOS	inducible nitric oxide synthase
INPPL1	Inositol Polyphosphate Phosphatase-Like 1 gene
JAK1/2	Janus kinase 1/2
JNK	c-Jun N-terminal kinase
KRAS	Kirsten rat sarcoma viral oncogene homolog
LPS	lipopolysaccharides
MALT	mucosa-associated lymphoid tissue
MAPK	Mitogen-Activated Protein Kinase
MEK1/2	Mitogen-activated protein kinase kinase 1 and 2
MET	MET proto-oncogene
Mfn1/2	mitofusin 1/2
MLH	mutL homolog 1
MLH1	MutL Protein Homolog 1
MnSOD	Manganese Superoxide Dismutase
MOMP	mitochondrial outer membrane permeabilization
mPTP	mitochondrial permeability transition pore
mRNA	messenger ribonucleic acid
MSH1	mutS homolog 1
MSH2	mutS homolog 2
MSH6	mutS homolog 6
MSI	microsatellite instability
mtBER	mitochondrial base excision repair
mtDNA	mitochondrial deoxyribonucleic acid
MTHFD2	Methylenetetrahydrofolate dehydrogenase (NADP+ dependent) 2
mtROS	mitochondrial reactive oxygen species
NADPH	Nicotinamide Adenine Dinucleotide Phosphate
NapA	neutrophil-activating protein
NEIL1/2	Nei-like DNA glycosylase 1
NFAT	nuclear factor of activated T cells
NFAT2	Nuclear Factor of Activated T cells 2
NFκB	nuclear factor kappaB
NLRP3	Nod-like receptor family pyrin domain-containing 3
NOX	NADPH oxidase
Nrf2	Nuclear factor erythroid 2-related factor 2.
NRLP3	NLR family pyrin domain-containing 3
ONOO-	peroxynitrite
OpA1	Optic Atrophy 1 protein
p33	p33 Mitogen-Activated Protein Kinase
p38	p38 Mitogen-Activated Protein Kinase
p55	p55 Mitogen-Activated Protein Kinase
p65/RelA	v-rel avian reticuloendotheliosis viral oncogene homolog A protein
PALB2	Partner and Localizer of BRCA2
PD-1	Programmed Cell Death Protein 1
PDL-1	Programmed Death-Ligand 1
PI(3,4)P	phosphatidilinositol 3,4 bisphosphate
PI(3,4,5)P	phosphatidilinositil 3,4,5 trisphosphate
PIK3CA	Phosphatidylinositol-4,5-Bisphosphate 3-Kinase Catalytic Subunit Alpha
PINK1	PTEN-induced kinase 1
POLB	polymerase beta
POLG	DNA polymerase gamma
PPARγ	Peroxisome Proliferator-Activated Receptor gamma
Prx/Trx	Peroxiredoxin/Thioredoxin antioxidant system
Prx2/Prx3	peroxiredoxin 2/peroxiredoxin 3
PtCo@G@H2A	dual-targeted cascade catalytic nanozyme PtCo@Graphene@Hemin-2(L-arginine)
PUMA 9	P53 Upregulated Modulator of Apoptosis
RAS/ERK/MAPK	Rat sarcoma/Mitogen-Activated Protein Kinase/Extracellular Signal-Regulated Kinase
RelA/IκBα	v-rel avian reticuloendotheliosis viral oncogene homolog A/Inhibitor of κB alpha
RelB/p52	v-rel avian reticuloendotheliosis viral oncogene homolog B/p52
RIRR	ROS-Induced ROS release
ROS	reactive oxygen species
RPTP β/α	Receptor-type Protein Tyrosine Phosphatase beta/alpha
RUNX3	Runt-related transcription factor 3
SabA	Sialic acid-binding adhesin
SHIP2	SRC homology 2 containing tyrosine phosphatase
SIRT1	Sirtuin 1
SIVA1	Apoptosis Regulatory Protein Siva-1
SMOX	spermine oxidase
SNU1	gastric cancer cell line
STAT3	Signal Transducer and Activator of Transcription 3
STAT3/YAP/β-catenin signaling	Signal Transducer and Activator of Transcription 3/Yes-associated protein/beta catenin signaling
STING	stimulator of interferon genes
t(1;14)(p22;q32) BCL10-IGH	B-cell lymphoma 10-immunglubuline heavy chain
t(11;18)(q21;q21) BCR3–MALT1	B-cell receptor 3- Mucosa-Associated Lymphoid Tissue Lymphoma Translocation Protein 1
t(14;18)(q32;q21) IGH-MALT1	immunglubuline heavy chain- Mucosa-Associated Lymphoid Tissue Lymphoma Translocation Protein 1
T4SS	type 4 secretion system
TDP1	tyrosyl-DNA ohosphodiesterase 1
TEER	transepithelial electrical resistance
TFAM	mitochondrial transcriptional factor A
TGF β	Transforming Growth Factor-beta
Th cells	T helper cells
TIM	translocase of the mitochondrial inner membrane
TLR4	toll like receptor 4
TNF α	tumor necrosis factor alpha
TNFR1	Tumor Necrosis Factor Receptor 1
TOM	translocase of the mitochondrial outer membrane
TP53	tumor protein 53
TRAIL	TNF-related apoptosis-inducing ligand
TRAIL-R1/R2	TNF-related apoptosis-inducing ligand receptor 1/2
Treg	regulatory T cells
ULF	E3 ubiquitn ligase
UPRmt	mitochondrial unfolded protein response
USP36-SOD2	Ubiquitin-specific Peptidase 36- Superoxide Dismutase 2
VacA	vacuolating cytotoxin A
VEGF	vascular endothelial growth factor
VEGFR	vascular endothelial growth factor receptor
YB-1	Y-box binding protein
YB-1	Y-box binding protein 1
